# Supplementary material for: Conceptual framework of applying CoI-based blended learning approach to enhance students’ vocabulary and vocabulary learning motivation
Source: PLoS One. 2025 Aug 12;20(8):e0330115. doi: 10.1371/journal.pone.0330115 (PMC12342318; doi:10.1371/journal.pone.0330115)
Supplement: S1 File — (DOCX) [file pone.0330115.s001.docx]

Appendices-

**Guidelines for the framework**

Table 1

Guidelines for conducting the CoI-based blended learning (Steps of designing learning procedure)

| **Procedure** | **Approach** | **Descriptions** |
| --- | --- | --- |
| **Preparations** | **--** | Before classes began, students were divided into distinct communities based on their performances and personalities. Online learning forums were also created on the ChaoXing Learning Platform according to the communities. In each community, students were allocated roles such as group leader, technical recorder, designer, and connector, and were responsible for a variety of learning-related tasks. |
| **Before class** | **Online** | **Information collection:** Individual and group assignments were assigned to students, who then met and discussed using the ChaoXing Learning Platform on blended distributed terminals to share the required and collected information for the class. |
|  |  | **Personal & group tasks:** The teacher provides various learning contents through the network, students complete their individual and group assignments using various digital media, such as their smartphones and laptops, at their preferred time, space, and speed and upload them to the ChaoXing Learning Platform according to the requirements of the pre-class tasks issued by the teacher. |
|  |  | **Discussions:** Students meet and discuss to share the collected information needed for the class and upload the required documents to the ChaoXing Learning Platform. |
|  |  | **Problems proposing:** Discuss and propose the problems they were facing when doing the pre-class individual and group assignments. |
| **During class**  **(face-to-face)** | **Online + offline** | **Presentations & lectures:** Volunteers and randomly chosen students by the ChaoXing Learning Platform were asked to present their work, and teachers gave them constructive and specific feedback. |
|  |  | **Discussions:** Students in a community or inter-communities together with the teacher discuss and explore the knowledge. |
|  |  | **Summaries:** The teacher summarizes the knowledge of the present class, emphasizing teaching key points and teaching difficult points. |
|  |  | **Evaluations:** Diversification of evaluations was done by students’ self-evaluation, inter-student evaluation, teacher evaluation, and automatic evaluation by the ChaoXing Learning Platform. |
| **After class** | **Online** | **Knowledge reflecting:** Students reflect on their strengths and weaknesses in the lesson’s knowledge based on their understanding, exploration, and the teacher’s summaries. |
|  |  | **Personal & group assignments:** Consolidation and deepening of knowledge through individual and group assignments after class based on what has been learned. |
|  |  | **Knowledge integrating:** Use diagrams, mind maps, knowledge comparisons, and knowledge points to organize and integrate what have learned in class. Further knowledge application, problem analysis, and solution exploration. |
|  |  | **Problems solving:** Through students’ self-exploration, peer discussion, and teachers’ explanation, eventually lead to the resolution of the confusing questions and problems. |

Table 2

Guidelines for conducting the CoI-based blended learning (Measures should be taken during learning procedure)

| **CoI elements** | **Theme** | **Descriptions** |
| --- | --- | --- |
| **Teaching presence** | **Guidance** | **Professional instructions:** Teachers need to comprehend the theory, belief, principle, and idea underlying teaching activities or methods. They need to master the relevant professional knowledge of teaching, such as teaching environment, curriculum, teaching design, teaching mode and method, classroom management, teaching evaluation, and teaching ability of information technology. Students’ needs, qualities, differences, and motivations need to be understood as well. |
|  |  | **Timely feedback:** The feedback should be expeditious, immediate, constructive, multiple, empathetic, critical, and original. Provide timely feedback on students’ in-class participation, correctness of their performances, online and offline learning scoring, and emotions. Feedback should be made to let students be involved in the classroom, learn more about their capabilities, and maintain their self-confidence. Provide students with the direction they require to achieve their objectives. |
|  |  | **Diversify evaluations:** To ensure the authenticity, objectivity, and real-time of the entire academic evaluation, an evaluation system that combines teacher evaluation, peer evaluation, and self-evaluation; online evaluation and offline evaluation; initial evaluation and summative evaluation; dynamic evaluation and static evaluation; standard regular evaluation and random flexible evaluation should be designed. |
|  | **Emotion** | **Behavior respecting:** Teachers respond to and appreciate students’ behaviors by trusting, supporting, smiling, making eye contact, employing calming postures, having a great sense of humor, being kind, using a decent tone, and sharing positive emotions. |
|  |  | **Confidence encouraging:** Encourage, praise, and reward acceptable, learning-oriented behaviors among students. Produce positive psychological hints to boost their self-confidence, and cohesion, reduce their anxiety, and encourage them to be courageous in expressing their opinions. |
|  |  | **Interest stimulating:** Create a fun and playful environment that enhances student’s motivation and learning outcomes by appealing to their interests. Motivate and stimulate their desire to learn and seek more knowledge. Allow students to focus more on their work and eliminate monotonous lecturing routines by using authentic and interesting materials and situations. |
| **Teaching presence** | **Supervision** | **Autonomous learning:** Enhance students’ capacity for self-directed learning, including self-monitoring and self-reflection. Teachers serve as mentors to students, assisting them in identifying learning objectives, defining content and progressions, selecting methods and strategies to be used, monitoring the acquisition process, and analyzing what has been learned. Help students create realistic goals so that when they achieve them. Let students feel a sense of achievement and develop the ability to learn on their own. |
|  |  | **Self-control ability:** This includes strong self-management skills, the ability to concentrate in class, refraining from cell phone use, distraction, and gossiping with classmates. The ability to accomplish group and individual assignments assigned by the teacher self-consciously. |
|  |  | **Continuous grit:** Students own perseverance, ambition, and energy for long-term objectives, and have a clear and profound understanding motivations and goals of their actions. Be capable of regulating their emotions in response to setbacks and failures, controlling their actions, and remaining unfazed and unruffled. |
| **Social presence** | **Interaction** | **Cooperative cooperation:** Provide a safe and comfortable classroom environment and avoid using threats. Encourage students to become active, imaginative, critical, and accountable in order to achieve the team’s objective. Let students participate in the learning experience in the entirety and effective group discussion while working in groups to provide a positive experience for every participant. |
|  |  | **Healthy competition:** In a healthy competition environment, as opposed to individuals trying to outperform one another, students work to achieve organizational and common objectives. Friendship comes before competition. |
|  |  | **Effective communications:** Construct communities of blended learning among instructors and students. Create a classroom environment that is beneficial, engaging, interactive, and pleasurable. Encourage synchronous and asynchronous discussion, effective communication, and benign interaction as well as questioning, responding, and commenting among students. |
| **Social presence** | **Affective collection** | **Teacher-students:** Teachers should be able to foster an environment in which students can interact freely and safely. Relationships between teachers and students in the classroom should be characterized by respect, enjoyment, mutual trust, and comprehension. |
|  |  | **Students-students:** Positive relationships between group members to boost social interaction, individual accountability, and group competence. Learn to be tolerant and empathetic, to care for others, to praise others, to respect and accept others, and to manage conflicts between classmates. |
|  |  | **Community-community:** Strong community spirit, prompt to assist, share resources and information, and respond to inquiries. Positive and effective interactions, active learning practices, knowledge, ideas, experience, and learning products are valued and shared among communities. |
|  | **Communication method** | **Diversify tools:** Apply digital technologies, such as computer-assisted language learning (CALL) or mobile-assisted language learning (MALL), digital games, learning management systems (LMS), learning platforms, and other digital tools such as Google Form, Socrative, Kahoot!, Quizizz, Quizlet, Youdao vocabulary learning app, Wechat those have the potential to enhance teaching and learning. |
|  |  | **Convenient platform:** When implementing the CoI-based blended learning, a user-friendly platform, such as the ChaoXing Learning Platform, Rain Classroom, Ke Tang Pai, Tencent Meeting, Google Classroom, Embodo, etc., can be used. |
|  |  | **Useful functions:** Functions such as Sign-in, Information sharing, Competitive answering, Vote, Quiz, Discussion, Random candidate, Assign homework, Class report, Big data analysis, Evaluation, and E-learning design for effective interactions between teachers and students or among students. |
| **Cognitive presence** | **Strategy** | **Digital resources:** Make full use of online digital resources like Chinese University MOOC, Smart VET, Secondary Teaching Resources Website, Technical Education Website, Chongqing Cloud Platform, and resources in the ChaoXing Learning Platform. Use fundamental mobile platforms to act as alternatives in low-tech regions. |
|  |  | **Novel tools:** Using new digital teaching tools that combine text, sound, and images to enrich classroom teaching, and improve the teaching quality of teachers and the learning efficiency of students. Use fundamental mobile platforms to act as alternatives in low-tech regions. |
|  |  | **Effective instructions:** Excellent instruction should be provided. Curriculum, books, software, and other instructional materials should make sense to students. Make them engage and feel easy to apply. The objectives of each course should be specified in the instructions with varying prior knowledge. Instructions should also include new subject matter, simple language, vivid images, and examples. |
|  | **Course approach** | **Immense learning:** A form of contextualized language learning in which the learning process is situated in a diversity of virtual or actual language contexts. In addition to the interaction with the teacher making the class vivacious, active, and challenging, the students are fully engaged and aware of the learning and processes taking place throughout the entire class period. |
|  |  | **Flipped classroom:** Classes are held in digital environments that promote the development of students’ competencies through cooperative learning, problem-solving, and critical thinking. An emphasis is additionally put on dynamic interaction and the transfer of knowledge using digital tools in a collaborative and idea-sharing spirit. Students learn various topics separately utilizing video tutorials before applying their knowledge in class by working on practical exercises. Students are encouraged to learn alone and in groups, which facilitates the exchange of learning content throughout communities and, as a result, improves students’ problem-solving abilities. |
|  |  | **Learning communities:** Students allocate to small groups to master academic content. Classroom discussions, presentations, and group projects are progressively transformed into collaborative learning through the use of technology. |
|  | **Meaningful learning** | **Curiosities arousal:** Piquing students’ motivation and curiosity about the material to be taught. For example, use surprising demonstrations, the comparison of subjects to students’ own lives, or allow students to find out information by themselves. |
|  |  | **Solutions digging:** Encourage students to establish and reinforce meaning by having ongoing discussions and reflections. Encouraging critical thinking in a community of students through emotional, interactive, and coherent responses to help reach cognitive learning objectives. |
|  |  | **Ideas exploration:** Sharing the knowledge and information gained through group discussions. Encourage critical thinking, problem-solving, and new ideas-creating abilities among students. |
