# Supplementary material for: Conceptual framework of applying CoI-based blended learning approach to enhance students’ vocabulary and vocabulary learning motivation
Source: PLoS One. 2025 Aug 12;20(8):e0330115. doi: 10.1371/journal.pone.0330115 (PMC12342318; doi:10.1371/journal.pone.0330115)
Supplement: Attachment — Submitted filename: Response to editor and reviewers.docx [file pone.0330115.s003.docx]

**Dear Editor and Reviewers,**

Thank you for your valuable comments on my manuscript entitled *Conceptual framework of applying CoI-based blended learning approach to enhance students’ vocabulary and vocabulary learning motivation* (PONE-D-25-04072). I have carefully addressed all suggestions and revised the paper accordingly. The key modifications are summarized in the second section below, which includes: (1) editor & reviewers’ comments, (2) author’s revision, and (3) corresponding page numbers for easy reference.

I believe these revisions significantly improve the manuscript’s clarity and rigor. Should further adjustments be needed, I am happy to refine the paper. Thank you again for your time and constructive feedback.

Sincerely,
Qiu Chuane

Email address: [2856126876@qq.com](mailto:2856126876@qq.com)

**Editor’s Comments**

- **Literature review**

**Comment 1-**Digital Transformation and Assessment: Consider incorporating recent perspectives on digital transformation in EFL to strengthen the rationale for your use of blended learning platforms. For instance:
- Al Fraidan & Alaliwi (2024). Digital Transformation for Sustainable English Language Learning
- Al Fraidan (2025). Evaluating Lexical Competency in Saudi Arabia’s Hybridized EFL Ecosystem

**Author’s revision**

**-P2**

“In recent years, the adoption of blended learning has surged, owing to its flexibility and the integration of digital tools that foster both independent and collaborative learning [1,5,6].”

**-P6**

“Recent study [73] underscores the need for adaptive and interactive assessment modalities to enhance both vocabulary acquisition and learner motivation.”

**Comment 2-**Learner Engagement and Motivation: The manuscript would benefit from integrating research on learner-centered practices in digital EFL contexts. In particular, the following work aligns closely with your study's emphasis on learner motivation and feedback responsiveness within digital pedagogies:
> Rasool, U., Qian, J. & Aslam, M. Z. (2024). Understanding the significance of EFL students’ perceptions and preferences of Written Corrective Feedback. Sage Open, 14(2), 1–11. [https://doi.org/10.1177/21582440241256562](https://doi.org/10.1177/21582440241256562)

**Author’s revision**

**-P28**

Align with [126,127], professional instruction, prompt feedback, varied evaluations, and consideration of students’ emotions cultivated students’ positive perceptions of Teaching Presence.

**Comment 3-**Theoretical Anchoring: Incorporate Self-Determination Theory (Deci & Ryan) as a motivational lens to further explain how CoI elements (especially social and cognitive presence) relate to autonomy, competence, and relatedness in learning.

**Author’s revision**

**-P4-6**

**Community of Inquiry and learning motivation**

Learning motivation influences students’ learning behaviors and guides them towards particular academic objectives [38]. Self-determination theory (SDT) posits that an individual’s desire to learn during an activity is contingent upon the extent to which the learning environment fulfills the three fundamental psychological requirements of autonomy, competence, and relatedness [39]. According to [40], these three fundamental psychological needs are significantly correlated with the three dimensions of the Community of Inquiry (CoI) framework: Teaching Presence (TP), Social Presence (SP), and Cognitive Presence (CP).

Comprehensive studies have established a substantial correlation between Teaching Presence (TP) and students’ motivation [41], especially within the context of Self-Determination Theory (SDT). Autonomy support is augmented when educators employ structured instructional frameworks, offer avenues for students’ choice, and promote self-directed learning [42]. By providing timely, substantive feedback and exhibiting passion, educators create an environment that empowers students to take initiative, thus nurturing intrinsic motivation [43]. Moreover, active listening, empathy, and personalized support enhance students sense of agency, consistent with autonomy as a fundamental catalyst for engagement [44]. Secondly, TP enhances competency via constructive feedback, explicit expectations, and scaffolded learning experiences [45]. When educators create engaged and intellectually stimulating sessions, students cultivate mastery beliefs and self-efficacy [46]. A well-organized educational setting, along with formative evaluations, allows students to monitor their advancement, hence enhancing their perceived competence [47]. Ultimately, TP enhances relatedness by cultivating a secure, inclusive, and cooperative classroom environment. Educators who demonstrate positive regard, accessibility, and emotional support foster a sense of value and connection among students [47]. The presence of social interaction in education, facilitated by responsive communication and community-building initiatives, improves relationships among peers and between instructors and students, thereby satisfying students demand for belonging [44]. This sense of connection is essential for enduring motivation, since it reduces disengagement and enhances emotional well-being [48].

Empirical research indicates that Social Presence (SP) is essential for satisfying learners’ psychological needs for autonomy, competence, and relatedness, thus augmenting intrinsic motivation and academic engagement [49]. First, SP enhances autonomy by promoting student-centered learning environments in which learners engage actively in decision-making and collaborative activities [50]. Students participating in peer discussions, group projects, or self-directed activities gain increased control over their learning, corroborating SDT’s claim that autonomy-supportive environments augment motivation [48]. Moreover, interactive and dialogic pedagogical approaches, including peer evaluations and collaborative problem-solving, enhance learners’ feeling of agency, diminishing dependence on external regulation and fostering self-determined behavior [49]. Second, the concept of competence is enhanced by SP, facilitating meaningful interactions that affirm learners’ capacities and support skill development [51]. Collaborative exercises, including group discussions and peer feedback sessions, enable students to assess their comprehension, enhance their knowledge, and obtain constructive feedback from peers and instructors. These interactions enhance subject-matter expertise and foster metacognitive and self-regulatory skills, vital for enduring academic achievement [52]. When educators facilitate supportive and organized social interactions, students cultivate enhanced self-efficacy, viewing themselves as competent participants in the learning community [53]. The most immediate impact of SP is on relatedness, since it fulfills the fundamental human need for connection and belonging [54]. A strong sense of community in educational settings enhances students emotional engagement and reduces feelings of isolation [55]. Small-group discussions and cooperative learning tasks, foster students’ trust and mutual support [55]. This interactive and socially enriched environments augment intrinsic motivation by validating students identities as legitimate participants in academic discourse [56].

Studies have repeatedly shown a significant beneficial correlation between Cognitive Presence (CP) and students’ motivation [57, 58]. CP promotes autonomy by facilitating self-directed inquiry and intellectual exploration [59]. Students participate in higher-order cognitive processes, including critical reflection and knowledge application, achieve enhanced congruence between their learning activities and personal values [60]. CP’s problem-solving aspect enables learners to exercise autonomous judgment, make significant decisions regarding their learning methodologies, and cultivate a sense of ownership over their educational experience [61]. The correlation between CP and competency is well-documented in the literature. By engaging consistently in critical discourse and using knowledge practically, students build subject-matter competence and metacognitive skills [62]. The iterative process of inquiry, reflection, and problem-solving intrinsic to CP offers ongoing chances for students to showcase and enhance their abilities [63]. When educational settings offer explicit learning objectives and formative assessment opportunities, students report increased emotions of competence and self-efficacy [64]. The predominantly cognitive aspect of persistent critical thinking and knowledge production also promotes relatedness via collaborative meaning-making [59]. The social aspect of CP arises during collaborative problem-solving tasks in which students participate in collective reflection and analysis [65]. This shared cognitive endeavor fosters a sense of intellectual community, fulfilling students need for belonging in academic environments [62]. The reflective elements of CP allow students to relate their learning experiences to wider social contexts, so augmenting their sense of purpose and affiliation with the learning community [57].

**-P27**

The **second sub-research question** indicated a notable increase in the experimental group’s Vocabulary Learning Motivation score of a 0.38-point improvement together with a medium effect size. This transition, as seen by [119], indicates a progression of students’ VLM from a Moderate level to a Motivated level. The enhancement of students’ VLM scores can be systematically elucidated by the CoI framework’s three presences which fostered students’ fundamental psychological needs of autonomy, competence, and relatedness [48]. First, by integrating well-crafted decision-making frameworks into blended learning, TP promoted students’ autonomy. In this study, TP offered autonomous support via three processes, in accordance with [120]: 1) Self-directed vocabulary modules enabling students to follow their own learning pace, 2) optional challenge tasks for advanced learners, and 3) customized learning path generated by the Chaoxing Learning APP algorithm. Competence was developed through the immediate feedback from the teacher and system. Recent neurocognitive studies indicate when learners proficiently acquire new vocabularies, their brain’s reward circuitry can be stimulated [121]. The varied assessment techniques in accordance with [122], such as peer evaluations and digital badges, established visible competence progression. It is essential for sustaining motivation in vocational students who frequently question their language learning capabilities. This study confirmed the findings of [123] that SP significantly affects learners’ EFL motivation, engagement, and overall learning outcomes in blended or online language learning environments. SP markedly improved relatedness by organized peer interactions. Collaborative contexts and peer interactions enhanced competence development through modeling, feedback, and shared challenges. This consequently enhanced VLM, as learners who perceived themselves as competent and socially supported demonstrated increased persistence [48]. In addition, digitally-mediated cooperation can overcome the social anxiety commonly found in vocational classrooms. CP enhanced all three psychological demands via cognitively stimulating language exercises. In alignment with [28] and [124], this CoI-based integrated learning environment offers a forum for students to exchange their perspectives on vocabulary acquisition and engage in critical discourse. Students can express difficulties, seek knowledge, comprehend essential issues through the integration of existing information, and subsequently formulate solutions to tackle those challenges. Autonomy was augmented by problem-based learning scenarios that permitted several solution pathways. The CP features unexpectedly enhanced relatedness, as students identified common difficulties and solutions during collaborative meaning-making activities [125]. Tasks were calibrated to balance challenge and skill, avoiding both oversimplification and excessive difficulty. These carefully scaffolded exercises fostered students’ competence. Metacognitive reflection exercises further targeted vocational students’ self-efficacy, with recent evidence showing such activities rebuild confidence in struggling learners. This sense of ownership and participation motivates students to engage more actively in their studies, in accordance with [81].

**Comment 4-**Wording Clarity: Sentences like “students were frequently provided with voluminous paper-based vocabulary books…” could be revised to:
> “Traditionally, students have relied on extensive paper-based vocabulary exercises, a method that often reduces engagement and limits contextualized learning opportunities.”

**Author’s revision**

**-P1**

“The mechanical traditional way of teaching and their lacking amount of vocabulary toward the target language often demotivate them in learning.”

>“The mechanical traditional way of teaching and their lack of vocabulary toward the target language often demotivate them in learning.”

**-P6**

“Students were frequently provided with voluminous paper-based vocabulary books and subjected to the laborious process of passive learning.”

>“Students have to rely on extensive paper-based vocabulary exercises, a method that often reduces engagement and limits contextualized learning opportunities. ”

**-P7**

“With this approach, students are offered a new learning experience and able to interact with digital platforms, extending the variety of available resources and addressing the needs of different learning styles with a combination of texts, images, audio, and videos (Tosun, 2015)”.

>“This strategy provides students with a novel learning experience. It enables interaction with digital platforms, broadening the array of resources, and accommodating diverse learning styles through a blend of texts, images, audio, and videos [75].”

...please refer to the tracked manuscript.

- **Methodology and Analysis**

**Comment 1-**Group Assignment and Sampling: Clarify how experimental and control groups were matched or controlled for equivalence. A sample addition:
> “Using purposive sampling, both groups were matched based on baseline vocabulary scores and demographics, with pre-test homogeneity confirmed (t(84) = 0.20, p = 0.83).”

**Author’s revision**

**-P8**

“Using purposive sampling, both groups were matched based on baseline vocabulary scores and demographics, with pre-test homogeneity confirmed (t (84) = 0.20, p = 0.83). ”

**Comment 2-**Mapping Intervention to CoI: While Table 1 lists CoI elements and activities, the manuscript would benefit from a visual flowchart or timeline mapping phases of the intervention explicitly to CoI constructs (e.g., group tasks = social presence; teacher feedback = teaching presence).

**Author’s revision**

**-P13-15**

Table 2. The Application CoI Framework- Take lesson *let’s go shopping* as an example

| **Teaching Steps** | **Teachers Activities** | **CoI Application** | **Students Activities** | **CoI Application** |
| --- | --- | --- | --- | --- |
| **Before- Class** | **Preview (Online)**  1.Create an online course and groups with the ChaoXing Learning App, and instruct students to log in using a QR code. | **Teaching Presence（TP）-** Design & Organization | **Preview (Online)**  1.Sign in the class and belonged group according to the invitation QR code. | -- |
|  | **Preview (Online)**  2.Release a short video -“shopping” to students on the ChaoXing Learning App:  <https://m.v.qq.com/play.html?vid=h07651mgg1c&cid=,> and ask them to finish preview task card 1 by group. | **Teaching Presence（TP）-** Design & Organization; Facilitating Discourse  **Social Presence（SP) -**  Affective Expression; Group Cohesion | **Preview (Online)**  2.Watch the short video- “shopping” and finish preview task card 1 by group. | **Cognitive Presence (CP)-** Triggering Event  **Social Presence（SP) -**  Affective Expression; Group Cohesion |
|  | **Preview (Online)**  3.Instruct students to read and preview the short reading in Exercise A on page P21, the important phrases in Exercise F on page P22, and the ten key words on page P164, and to complete preview task card 2 individually. | **Teaching Presence（TP）-** Design & Organization | **Preview (Online)**  3.Complete the preview tasks of the short reading in Exercise A on page P21, Key sentences in Exercise F on page P22, and 10 key words on page P164 and finish preview task card 2 personally. | **Cognitive Presence (CP)-** Triggering Event |
|  | **Preview (Online)**  4.Instruct students to research Martins Department Store, 24-7 Convenience Store, Sams Food Market, Hero Books, Shoe Emporium, Dress for Less, and Thrift Central, and complete preview task card 3 collaboratively. | **Teaching Presence（TP）-** Design & Organization; Facilitating Discourse  **Social Presence（SP) -**  Affective Expression; Open Communication; Group Cohesion | **Preview (Online)**  4.Investigate details regarding Martins Department Store, 24-7 Convenience Store, Sams Food Market, Hero Books, Shoe Emporium, Dress for Less, and Thrift Central, then complete the preview task card 3 collaboratively as a group. | **Cognitive Presence (CP)-** Triggering Event  **Social Presence（SP) -**  Affective Expression; Open Communication;  Group Cohesion |
|  | **Preview (Online)**  5.Release a micro lesson on the use of lexical verbs in the simple present tense: https://b23.tv/0oOhiL6 Instruct students on the ChaoXing Learning App to collaboratively complete preview task card 4. | **Teaching Presence（TP）-** Design & Organization; Facilitating Discourse  **Social Presence（SP) -**  Affective Expression; Open Communication; Group Cohesion | **Preview (Online)**  5.Watch the micro lesson of lexical verb used in Simple present tense: <https://b23.tv/0oOhiL6,> and finish preview task card 4 by group. | **Cognitive Presence (CP)-** Triggering Event  **Social Presence（SP) -**  Affective Expression; Open Communication; Group Cohesion |
|  | **Preview (Online)**  6.Ask students to upload preview tasks cards to the ChaoXing Learning App and do the self-assessment of preview tasks. | **Teaching Presence（TP）-** Design & Organization | **Preview (Online)**  6.Finish preview task cards and upload them to the ChaoXing Learning App and do the self-assessment of preview tasks. | -- |
| **In-Class** | **Lead-in (Online+Face-to-face)**  1.Issues a sign-in gesture on the ChaoXing Learning App. **(1min)** | **Teaching Presence（TP）-** Design & Organization | **Lead-in (Online+Face-to-face)**  1. Sign-in through the ChaoXing Learning App by gesture. | -- |
|  | **Lead-in (Online+Face-to-face)**  2.Present students with images related to shopping and pose the question: Do you enjoy shopping? Request students to generate and list the vocabulary related to shopping on the screen. Present the subject of the current lesson.  **(2min)** | **Teaching Presence（TP）-** Design & Organization; Facilitating Discourse  **Social Presence（SP) -**  Affective Expression; Open Communication | **Lead-in (Online+Face-to-face)**  2.Respond to the instructor’s inquiries. Generating and listing the vocabulary related to shopping that they are familiar with on the screen. | **Cognitive Presence (CP)-** Triggering Event  **Social Presence（SP) -**  Affective Expression; Open Communication |
|  | **Vocabulary Learning (Online+Face-to-face)**  3.Read about Van on page 21 with students and introduce Van’s problem to students. **(2min)** | **Teaching Presence（TP）-** Design & Organization; Facilitating Discourse  **Social Presence（SP) -**  Affective Expression; Open Communication | **Vocabulary Learning (Online+Face-to-face)**  3.Read about Van on page 21with the teacher and get to know Van’s problem | **Cognitive Presence (CP)-** Triggering Event  **Social Presence（SP) -**  Affective Expression; Open Communication |
|  | **Vocabulary Learning (Online+Face-to-face)**  4.Explain 10 key words and 1 sentence pattern of this lesson. **(8min)** | **Teaching Presence（TP）-** Design & Organization; Facilitating Discourse; Direct Instruction  **Social Presence（SP) -**  Affective Expression; Open Communication. | **Vocabulary Learning (Online+Face-to-face)**  4.Learn 10 key words and 1 sentence pattern of this lesson. | **Cognitive Presence (CP)-** Exploration  **Social Presence（SP) -**  Affective Expression;  Open Communication. |
|  | **Vocabulary Learning (Online+Face-to-face)**  5.Release the Seewo words game through the ChaoXing Learning App and announce the rules to the students. **(8min)** | **Teaching Presence（TP）-** Design & Organization; Facilitating Discourse  **Social Presence（SP)-**  Open Communication; Group Cohesion | **Vocabulary Learning (Online+Face-to-face)**  5.Each group selects one representative to compete in pairs, with the victor remaining until the conclusion of the game. | **Cognitive Presence (CP)-** Exploration  **Social Presence（SP)-**  Open Communication;  Group Cohesion |
|  | **Vocabulary Learning**  **(Online+Face-to-face)**  6.Select students at random to present the information they gathered on various stores before class.  **(6min)** | **Teaching Presence（TP）-** Design & Organization; Facilitating Discourse  **Social Presence（SP) -**  Affective Expression; Open Communication; Group Cohesion | **Vocabulary Learning (Online+Face-to-face)**  6.Present the information about different stores that they collected before class by group. | **Cognitive Presence (CP)-** Exploration  **Social Presence（SP) -**  Affective Expression;  Open Communication; Group Cohesion |
|  | **Vocabulary Learning (Online+Face-to-face)**  7.Ask students to help Van find the right stores to buy her needed items and finish Exercise B & C. **(5min)** | **Teaching Presence（TP）-** Design & Organization; Facilitating Discourse; Direct Instruction  **Social Presence（SP) -**  Affective Expression; Open Communication | **Vocabulary Learning (Online+Face-to-face)**  7.Help Van find the right stores to buy her needed items and finish Exercise B & C. | **Cognitive Presence (CP)-** Exploration  **Social Presence（SP) -**  Affective Expression;  Open Communication |
|  | **Vocabulary Learning (Online+Face-to-face)**  8.Provide directives for students to conduct Exercise D: Listen to Van and her spouse. Identify and circle the best location to get each item.  **(3min)** | **Teaching Presence（TP）-** Design & Organization; Facilitating Discourse; Direct Instruction  **Social Presence（SP) -**  Affective Expression; Open Communication. | **Vocabulary Learning (Online+Face-to-face)**  8.Complete Exercise D: Listen to Van and her husband. Circle the best place to get each item. | **Cognitive Presence (CP)-** Exploration  **Social Presence（SP) -**  Affective Expression;  Open Communication. |
|  | **Conversation Practicing (Online+Face-to-face)**  9.Instruct students to utilize the Youdao Learning App to capture images of the sentence pattern from Exercise F on page 22 and subsequently read aloud using the App.  **(5min)** | **Teaching Presence（TP）-** Design & Organization; Facilitating Discourse | **Conversation Practicing (Online+Face-to-face)**  9.Utilize the Youdao Learning App to capture images of the phrase structure from Exercise F on page 22 and subsequently read them using the App. | **Cognitive Presence (CP)-**  Exploration |
|  | **Conversation Practicing (Online+Face-to-face)**  10.Students are randomly chosen via the “shake” function of the ChaoXing Learning App to construct phrases incorporating “shoes”, “shirts”, “dictionary”, “bread”, “cheese”, and “fruit” within new dialogues utilizing the acquired sentence structure.  **(7min)** | **Teaching Presence（TP）-** Design & Organization; Facilitating Discourse  **Social Presence（SP) -**  Affective Expression; Open Communication | **Conversation Practicing (Online+Face-to-face)**  10.The chosen students construct phrases using “shoes,” “shirts,” “dictionary,” “bread,” “cheese,” and “fruit” within new dialogues utilizing the learnt sentence structure. | **Cognitive Presence (CP)-**  Exploration  **Social Presence（SP) -**  Affective Expression;  Open Communication |
|  | **Grammar Tips**  **(Online+Face-to-face)**  11.Facilitate a theme discourse on the ChaoXing Learning App and instruct students to analyze, deliberate, and synthesize the use of the term “shop” in various phrases within Exercise G: Examine the chart alongside your classmates and instructor.  **(5min)** | **Teaching Presence（TP）-** Design & Organization; Facilitating Discourse  **Social Presence（SP) -**  Affective Expression; Open Communication | **Grammar Tips**  **(Online+Face-to-face)**  11.Refer to Exercise G: Engage in a debate regarding the teacher’s inquiry and submit your response on the ChaoXing Learning App via theme discourse. | **Cognitive Presence (CP)-**  Exploration  **Social Presence（SP) -**  Affective Expression;  Open Communication |
|  | **Grammar Tips**  **(Online+Face-to-face)**  12.Provide more instances of lexical verbs utilized in the simple present tense and instruct students to summarize and conclude. Exercise H: Fill in the sentences with the appropriate form of the verb “shop”.  **(6min)** | **Teaching Presence（TP）-** Design & Organization; Direct Instruction | **Grammar Tips**  **(Online+Face-to-face)**  12.Summarize the application of lexical verbs in the simple present tense and conclude. Exercise H: Fill in the sentences with the appropriate form of the verb “shop”. | **Cognitive Presence (CP)-**  Exploration |
|  | **Summary**  **(Online+Face-to-face)**  13.Distribute an online survey via the ChaoXing Learning App and request students to create a bar graph to analyze the frequency of classmates purchasing at various types of Chinese retailers by group.  **(8min)** | **Teaching Presence（TP）-** Design & Organization; Facilitating Discourse  **Social Presence（SP) -**  Affective Expression; Open Communication; Group Cohesion | **Summary**  **(Online+Face-to-face)**  13.Complete the online survey and create a bar graph to analyze the frequency of classmates shopping at various types of Chinese retailers by group. | **Cognitive Presence (CP)-**  Integration  **Social Presence（SP) -**  Affective Expression;  Open Communication;  Group Cohesion |
|  | **Summary**  **(Online+Face-to-face)**  14.Review, summarize and fill in the mind map with knowledge learned in this lesson together with students. **(7min)** | **Teaching Presence（TP）-** Design & Organization; Facilitating Discourse; Direct Instruction  **Social Presence（SP) -**  Affective Expression; Open Communication | **Summary**  **(Online+Face-to-face)**  14.Review, summarize and fill in the mind map with knowledge learned in this lesson together with the teacher. | **Cognitive Presence (CP)-**  Integration  **Social Presence（SP) -**  Affective Expression;  Open Communication |
|  | **Evaluation**  **(Online+Face-to-face)**  15.Facilitate students in completing the self-assessment form, peer evaluation form, and teacher evaluation form via the ChaoXing Learning App.  **(5min)** | **Teaching Presence（TP）-** Design & Organization; Facilitating Discourse; Direct Instruction  **Social Presence（SP) -**  Affective Expression; Open Communication; Group Cohesion | **Evaluation**  **(Online+Face-to-face)**  15.Complete the students self-evaluation form, peer evaluation form through the ChaoXing Learning App. | **Cognitive Presence (CP)-**  Integration  **Social Presence（SP) -**  Affective Expression;  Open Communication;  Group Cohesion |
| **After- class** | **Expanding (Online)**  1.Discharge Active Task. Visit a shopping mall or utilize the Internet. Identify the names of three apparel retailers that you favor. Prepare a report and post it to the ChaoXing Learning App as a group. | **Teaching Presence（TP）-** Design & Organization; Facilitating Discourse; Affective Expression  **Social Presence（SP)-**  Open Communication; Group Cohesion | **Expanding**  **(Online)**  1.Execute Active Task. Visit a shopping mall or utilize the Internet. Identify the names of three apparel retailers that you favor. Prepare a report and post it to the ChaoXing Learning App as a group. | **Cognitive Presence (CP)-**  Resolution  **Social Presence（SP) -**  Affective Expression;  Open Communication;  Group Cohesion |
|  | **Expanding (Online)**  2.Instruct students to complete pertinent exercises in their exercise books and upload photographs of them to the ChaoXing Learning App. | **Teaching Presence（TP）-** Design & Organization | **Expanding**  **(Online)**  2.Finish relevant exercises on exercise book and upload it to the ChaoXing Learning App by photos. | **Cognitive Presence (CP)-**  Resolution |
|  | **Expanding (Online)**  3.Ask students to finish vocabulary exercise on the ChaoXing Learning App. | **Teaching Presence（TP）-** Design & Organization | **Expanding**  **(Online)**  3.Finish vocabulary exercise on the ChaoXing Learning App. | **Cognitive Presence (CP)-**  Resolution |
|  | **Evaluation (Online)**  4.Assess students’ homework and prompt them to conduct self-evaluations of their extracurricular assignments. | **Teaching Presence（TP）-** Design & Organization | **Evaluation (Online)**  4.Do self-assessment about their after-class tasks. | **--** |

**Comment 3-**Instrumentation: Further details on validation of the vocabulary test and VLM questionnaire (e.g., pilot testing, expert review) would improve rigor.

**Author’s revision**

**-P9**Data collecting and analysis

“Prior to the main study, a pilot study was conducted to obtain feedback regarding the reliability of the instruments [99].”

**-P8** Vocabulary test

“Following the creation of the draft, it was subjected to evaluation and approval by a panel of two experts in Chinese secondary vocational EFL education. ”

**-P9** VLM questionnaire

“Initially comprising 20 items, it was subsequently refined to 19 items following the two aforementioned experts evaluation due to redundancy in the descriptions of two questions. ”

**Comment 4-**Qualitative Coding: Thematic/categorisation analysis using open, axial, and selective coding is well-described. Consider providing one example of a coding decision, for instance, you should make a section 'Trustwothiness', (please see for theoratical understanding of Trustworthiness Graneheim, U. H., & Lundman, B. (2004). Qualitative content analysis in nursing research: concepts, procedures and measures to achieve trustworthiness. Nurse Education Today, 24(2), 105-112. [https://doi.org/10.1016/j.nedt.2003.10.001](https://doi.org/10.1016/j.nedt.2003.10.001) and to add recent reference please see section 4.2 Trustworthiness of Alyaqoub, R., Alsharairi, A. & Aslam, M. Z. (2024). Elaboration of Underpinning Methods and Data Analysis Process of Directed Qualitative Content Analysis for Communication Studies. Journal of Intercultural Communication, 24(2), 108-116. [https://doi.org/10.36923/jicc.v24i2.573](https://doi.org/10.36923/jicc.v24i2.573) ) and mention whether inter-coder reliability was checked or why not.

**Author’s revision**

**-P10-11**

Conversely, qualitative data were analyzed via thematic analysis. According to [101], to achieve trustworthiness, selecting individuals with diverse backgrounds enhances the probability of providing insight into the study subject from multiple perspectives. Therefore, the interviewees were students of four high proficiency level, four medium proficiency level, and four low proficiency level students according to their Vocabulary Test scores. Initially, written transcripts were generated from the interview recordings. The transcripts were thereafter printed and provided to the interviewers for verification of their alignment with the original meaning. Subsequently, the Chinese interview data was translated into English and submitted to a translator for verification. Inter-coder reliability assessments have been suggested to enhance reliability by evaluating concordance among several coders [102]. According to Miles and Huberman [103]’s guidelines for two researchers working together on qualitative analysis. Thematic analysis was completed by the researcher and teacher A (a lecturer in TESOL with more than ten years of teaching experience). The coding process follows the Open, Axial, and Selective Coding approaches by [104]. Codes were generated to satisfactory levels after the researcher and teacher A’s discussions and clarifications. Upon the conclusion of the three procedures, two Chinese experts in Chinese vocational EFL teaching field reviewed the coding process and provided suggestions concerning the emerging themes and sub-themes.

**Table 1. Coding example of interview transcripts**

| **Questions & Answers** | **Open coding** | **Axial coding** | **Selective coding (Sub-themes)** | **Themes** |
| --- | --- | --- | --- | --- |
| S1-Q3: Did you feel motivated to learn vocabulary in this CoI-based blended learning course?  A: *I think my motivation for learning is pretty good because, in this way, we can be more engaged. Then sometimes the teacher uses some encouragement methods. For example, every week the teacher evaluates the top three students in the system and rewards them. This makes us more motivated to learn. In addition, I think the Chaoxing Learning app is very practical and useful, there are so many functions and these functions brought us fun activities.* | 1)The teacher uses some encouragement methods which made the students more motivated to learn;  2)The Chaoxing Learning app is very practical and useful with many functions.  3)Chaoxing Learning app provides the students fun activities. | 1)Teachers take encouraging measures;  2)The platform has practical and useful functions;  3)Fun activities taken to motivate students; | 1)Confidence encouraging;  2)Useful functions;  3)Interest stimulating; | 1)Emotion;  2)Communication method; |
| S2-Q4: What do you think the strengths and weaknesses of vocabulary acquisition are in this CoI-based blended learning course?  *A: The disadvantage is that secondary school students are more or less unconscious, although they are usually very active in the classroom, there are inevitably a few students who take the opportunity to browse other websites when having class. Another disadvantage is that we have to do the homework on the mobile phone, and some of the answers can be found directly online. Some students will just copy and paste it over without using their brains at all. The advantage is that it can improve the enthusiasm of students because mobile phones have a youthful nature. For students of our age, being able to get along with mobile phones will make us very excited, and then those students who are sleeping can be effectively stopped.* | 1)A few students browsed other websites during the class;  2)Some students just copy and paste the answers online;  3)This CoI-based blended learning approach improved students’ enthusiasm;  4)Mobile phones made students excited. | 1)Students autonomous learning is not good;  2)Students self-control ability should be enhanced; | 1)Autonomous learning;  2)Self-control ability; | 1)Supervision; |
| S3-Q5: What do you think of the online and offline discussion in this CoI-based blended learning environment?  *A:I feel that in this blended learning environment, there is much more interaction between the teacher and the students and between the students themselves than in the traditional classroom. This mutual interaction is very helpful to enhance the relationship between the students and also between the teacher and the students. Students often discuss and solve problems proactively and enthusiastically online and offline together. They do not evade responsibility. Everyone participates in discussions actively, voluntarily, and proactively.* | 1)There is much more interaction between the teacher and the students and between the students themselves in this CoI-based blended learning environment;  2)Mutual interaction is very helpful in enhancing the relationship between the students and also between the teacher and the students;  3)Students often discuss and solve problems proactively and enthusiastically online and offline together. | 1)Interactions occurred among the teacher and students;  2)The relationship between the teacher and students was good;  3)Students solve problems actively. | 1)Effective communications;  2)Teacher-students, Students-students;  3)Solutions digging. | 1)Interaction;  2)Affective collection;  3)Meaningful learning |

**Comment 5-**Statistical Assumptions: Add notes on testing for normality (e.g., Shapiro–Wilk) and clarify whether assumptions for parametric tests were met.

**Author’s revision**

**-P16**

Thode [105] claims that the Shapiro-Wilk test is the optimal method for assessing normality in data when sample sizes are below 50. Upon analyzing the p-values in the Sig. column of the Shapiro-Wilk tests presented in Table 5, it is evident that all p-values exceeded 0.05. Therefore, the distributions of the Vocabulary pre-test and post-test scores, as well as the VLM pre-intervention and post-intervention scores for both the experimental and control groups, were normally distributed. The parametric paired samples t-test and independent samples t-test can be proceeded.

**Table 5. Normality test results for VTS and VLM measures by group**

| **Group** | **Item** | **Shapiro-Wilk** | | |
| --- | --- | --- | --- | --- |
|  |  | **Statistic** | **df** | **Sig.** |
| **Experimental group** | VTS-pre | 0.951 | 44 | 0.059 |
|  | VTS-post | 0.949 | 44 | 0.052 |
|  | VLM-pre | 0.97 | 44 | 0.297 |
|  | VLM-post | 0.958 | 44 | 0.111 |
| **Control group** | VTS-pre | 0.952 | 42 | 0.073 |
|  | VTS-post | 0.955 | 42 | 0.100 |
|  | VLM-pre | 0.984 | 42 | 0.818 |
|  | VLM-post | 0.979 | 42 | 0.641 |

**VTS: Vocabulary test scores; VLM:Vocabulary learning motivation;*

**Comment 6-**Effect Size Interpretation: Though eta squared is reported, please explain its educational significance. For example, what does η² = 0.232 mean in practice for vocabulary learning outcomes?

**Author’s revision**

The author recalculated the effect size via Cohen’s d and Cohen’s d confidence interval. The effect size has been discussed in the discussion part.

**-P10**

Cohen’s d and the Confidence Interval were used to compute the effect size. The formula for Cohen’s d is:

Where: 1) *M_1_* and *M_2_* are the means of the two groups being compared. 2) *SD_1_* and *SD_2_* are the standard deviations of the two groups. The standard error of Cohen’s d was used to calculate the confidence intervals. The formula for the standard error is:

Where: *n_1_* and *n_2_* are the sample sizes of the two groups. The 95% confidence intervals for Cohen’s d were calculated using the standard error. The formula for the confidence intervals is:

CI = *d* ± 1.96 × *SE_d_*

Where: 1) *d* is the calculated Cohen’s *d*. 2) SE*d* is the standard error of Cohen’s *d*. 3) 1.96 is the z-score corresponding to a 95% confidence level (for a two-tailed test).

**-P26-30**

The author has rewritten the discussion part. Please refer to the author’s revision text in the following section: Results and Discussions- Comment 1. or refer to the tracked manuscript.

- **Results and Discussion**

**Comment 1-**Educational Impact of Gains: Elaborate on what a ~9-point gain in VTS means pedagogically.

**Author’s revision**

**-P26-30**

The author has rewritten the discussion part. The effect size has been discussed as follows:

This study sought to examine the effect of a Community of Inquiry (CoI)-based blended learning approach on students’ Vocabulary Test Scores (VTS) and Vocabulary Learning Motivation (VLM) in Chinese secondary vocational EFL classrooms. The results demonstrated substantial enhancements in both VTS and VLM for students subjected to the CoI-based blended learning method in contrast to those in the conventional face-to-face learning setting. Furthermore, students exhibited favorable perceptions of this CoI-based blended learning approach. This study advances prior research by demonstrating how certain attributes of Teaching Presence (TP), Social Presence (SP), and Cognitive Presence (CP) affect students’ VTS and VLM in vocational blended learning contexts.

The findings from the first sub-research question indicated an approximately 9-point vocabulary gain with a large effect size. Chinese secondary vocational students generally start with vocabulary sizes 800-1,000 words beneath the national secondary education benchmark. Conventional vocabulary teaching methods have faced criticism for their excessive dependence on rote memorization and decontextualized word lists. Therefore, this significant enhancement is especially remarkable within the realm of Chinese secondary vocational education. The success of the VTS intervention can be systematically elucidated using the three components of the CoI framework. The TP component was implemented via meticulously crafted digital scaffolding that tackled essential issues in vocational EFL training. Consistent with previous studies [104,105,14,8], the instructor’s well organized lesson plans, incorporating pre-class previews, in-class exercises, and post-class reinforcement, guaranteed that students got scaffolded vocabulary training. The multimodal vocabulary presentation, which integrated video, text, and audio, boosted students’ involvement and facilitated students’ transition from rote memory to profound cognitive thinking [107]. Furthermore, interviewees indicated that the instructor’s feedback markedly enhanced their confidence. This corresponds with [108], indicating that the teacher’s role in facilitating discourse, delivering immediate feedback, and controlling group interactions assisted students in consolidating language. Social Presence was similarly vital to the success of the intervention, especially in tackling the motivating issues common in vocational EFL settings. This research emphasizes the critical role of fostering a collaborative online learning community to enhance students’ performance, consequently improving their engagement, consistent with the findings of [109,110,111]. This addressed as the principal impediment for vocational students: lack of motivation and engagement rather than cognitive incapacity. Interviews indicated that students had greater experiences of posing questions online compared to face-to-face classroom settings. That was because the CoI-based blended learning environment enabled students to share feelings, challenges, and achievements, thereby cultivating a supportive environment [27]. Such feeling of community is vital for enabling students to feel welcomed, supported, and accepted, hence increasing their VTS. These conclusions are consistent with the findings of [112-114]. CP was developed through well-organized exercises that fostered advanced engagement with vocabularies. According to [115], CP improves learner interest and active participation in online EFL discussions. Community members were urged to participate in peer interaction, problem-solving, inquiry, proposing solutions, and collaboration, thereby cultivating a flexible learning environment. Problem-based scenarios necessitated the contextual application of newly acquired vocabulary, fostering the desirable difficulty that has been demonstrated to improve long-term memory [116]. Metacognitive reflection exercises, in which students recorded and examined their vocabulary acquisition techniques, leveraged recent findings indicating that explicit strategy instruction can significantly aid vocational learners [117]. Furthermore, the components of the flipped classroom facilitated enhanced cognitive engagement during in-person sessions, aligning with recent research indicating that prior digital exposure liberates cognitive resources for advanced application in face-to-face classrooms [118].

The second sub-research question indicated a notable increase in the experimental group’s VLM score of a 0.38-point improvement together with a medium effect size. This transition, as seen by [119], indicates a progression of students’ VLM from a Moderate level to a Motivated level. The enhancement of students’ VLM scores can be systematically elucidated by the CoI framework’s three presences which fostered students’ fundamental psychological needs of autonomy, competence, and relatedness [48]. First, by integrating well-crafted decision-making frameworks into blended learning, TP promoted students’ autonomy. In this study, TP offered autonomous support via three processes, in accordance with [120]: 1) Self-directed vocabulary modules enabling students to follow their own learning pace, 2) optional challenge tasks for advanced learners, and 3) customized learning path generated by the Chaoxing Learning APP algorithm. Competence was developed through the immediate feedback from the teacher and system. Recent neurocognitive studies indicate when learners proficiently acquire new vocabularies, their brain’s reward circuitry can be stimulated [121]. The varied assessment techniques in accordance with [122], such as peer evaluations and digital badges, established visible competence progression. It is essential for sustaining motivation in vocational students who frequently question their language learning capabilities. This study confirmed the findings of [123] that SP significantly affects learners’ EFL motivation, engagement, and overall learning outcomes in blended or online language learning environments. SP markedly improved relatedness by organized peer interactions. Collaborative contexts and peer interactions enhanced competence development through modeling, feedback, and shared challenges. This consequently enhanced VLM, as learners who perceived themselves as competent and socially supported demonstrated increased persistence [48]. In addition, digitally-mediated cooperation can overcome the social anxiety commonly found in vocational classrooms. CP enhanced all three psychological demands via cognitively stimulating language exercises. In alignment with [28,124], this CoI-based integrated learning environment offers a forum for students to exchange their perspectives on vocabulary acquisition and engage in critical discourse. Students can express difficulties, seek knowledge, comprehend essential issues through the integration of existing information, and subsequently formulate solutions to tackle those challenges. Autonomy was augmented by problem-based learning scenarios that permitted several solution pathways. The CP features unexpectedly enhanced relatedness, as students identified common difficulties and solutions during collaborative meaning-making activities [125]. Tasks were calibrated to balance challenge and skill, avoiding both oversimplification and excessive difficulty. These carefully scaffolded exercises fostered students’ competence. Metacognitive reflection exercises further targeted vocational students’ self-efficacy, with recent evidence showing such activities rebuild confidence in struggling learners. This sense of ownership and participation motivates students to engage more actively in their studies, in accordance with [81].

The results from the second research question indicated that students have a favorable and preferred perception of this CoI-based blended learning approach. The advantages of this CoI-based blended learning approach were revealed nine themes and 27 sub-themes from TP, SP and CP. In line with [111], when educators are adept at using technology resources, students’ learning experiences are markedly enhanced. Align with [126, 127], professional instruction, prompt feedback, varied evaluations, and consideration of students’ emotions cultivated students’ positive perceptions of TP. Furthermore, in accordance with [82] trust was fostered through collaboration, communication, and dialogue, which enhanced the development of confidence in self-expression. Students and teachers can openly discuss and express their authentic feelings through various channels, including online forums on the Chaoxing Learning App and face-to-face interactions. All these enable students’ positive perceptions of SP. Moreover, the metacognitive components of this CoI-based blended learning environment effectively cultivated students’ curiosity, strategic awareness and problem-solving skills [128], the enhanced CP helped address the recognized deficiency in the learning skills of vocational students.

Notwithstanding the favorable opinions expressed by the students, the implementation of this CoI-based blended learning approach in practical teaching also poses challenges. For example, students struggled to maintain sustained concentration during class. They also faced difficulties resisting distractions, particularly from mobile phone usage. Additionally, many found it challenging to avoid off-task conversations with peers. These issues were linked to underdeveloped self-regulation and autonomy skills. Therefore, students suggested that the teacher should give more supervision during the learning process in the future. In the same way, past studies [129-131] all noted that structured and supportive learning environments contribute to better academic resilience. Teachers ought to provide guiding roles by offering helpful feedback, impartial observations, and constructive views during tasks. Furthermore, students noted that the Chaoxing Learning App is inadequate for facilitating CoI-based blended learning. Future learning processes require a variety of supplementary digital tools that are engaging, valuable, user-friendly, and effective for interaction with instructors, peers, and content. In this way, instructors are able to develop more dynamic, efficient, and equitable language learning experiences [90]. Besides, some students believed that certain functions of the Chaoxing Learning App might be enhanced to increase its convenience. In the future, it should facilitate students’ access to and download of courseware and instructional materials from any location at any time, while also allowing them to structure their own learning process. To address this problem, [73] suggested that the use of AI-driven platforms can facilitate more tailored and adaptive assessment experiences that correspond with the requirements of a contemporary, blended educational framework. What’s more, the CoI-based blended learning approach also faces challenges in low-tech vocational settings. Dindar et al.’s [132] suggestion of using fundamental mobile platforms to act as alternatives to more advanced systems can be considered. For example, in high-tech regions, AI-powered tutoring system and VR simulations can be applied; however, in low-tech areas, We-chat group and Tencent QQ group can be used instead.

**Comment 2-**Qualitative Summary Table: Include a table linking themes to representative student quotes to enhance clarity and synthesis.

**Author’s revision**

**-P21**

Table 14. Themes related to teaching presence

| **Teaching Presence** | **Guidance** | A) **Professional**   **Instruction** | *I could feel that the teacher had prepared her class more carefully in this blended learning environment. The teacher incorporated a lot of* ***professional knowledge*** *related to the subject of English. It is not as casual as traditional teaching.*  (S5) |
| --- | --- | --- | --- |
|  |  | B) **Timely**   **Feedback** | *We used to have to wait a long time for the teacher to finish correcting our assignments and papers after exams, but with the blended learning model, we can know where we* ***went wrong in time*** *and make targeted corrections.*  (S10) |
|  |  | C) **Diverse**   **Evaluations** | ***Peer assessment*** *is one of my favorite classroom activities, and whenever the teacher distributes students work, the classroom can be somewhat active.*  (S4) |
|  | **Emotion** | A) **Behaviour**   **respecting** | *In traditional education, teachers may be reluctant to ask students with low grades to answer questions. But in the blended learning approach, independent of our grades, we can engage in quizzes, brainstorming, topic discussions, group projects, etc. I feel that everyone* ***received adequate attention****.*  (S9) |
|  |  | B) **Confidence**   **encouraging** | *In the past, I was afraid of answering the teacher’s questions because I was afraid that I would make mistakes, but in this blended learning environment, I am active to challenge myself because it is really* ***encouraging from the teachers side****.*  (S12) |
|  |  | C) **Diverse**   **Interest stimulating** | *In this blended teaching mode, everyone is more active and* ***interested especially in learning vocabulary****, and then, in traditional teaching like in junior middle school, the teacher usually follows the book and speaks in a very rigid way, and then you tend to get distracted in class.*  (S3, S4, S6) |
|  | **Supervision** | A) **Autonomous**   **learning** | *As secondary school students are more or less unconscious, although they are usually very active in the classroom, there are inevitably a few students who take the opportunity to* ***browse other websites*** *when having class.*  (S2) |
|  |  | B) **Self-control**   **ability** | *Sometimes group members would* ***browse other websites*** *not related to the class, therefore, it is necessary for us to gain the teachers supervision.*  (S8) |
|  |  | C) **Diverse**   **Continuous grit** | *I feel that learning English is a long-term process, and because our classmates have all been rather disinterested in the past, I was concerned that they would find it* ***difficult to continue*** *once the novelty wore off.*  (S5) |

**-P23**

Table 15. Themes related to social presence

| Teaching Presence | **Interaction** | **A) Cooperative Collaboration** | *I could feel that the teacher had prepared her class more carefully in this blended learning environment. The teacher incorporated a lot of* ***professional knowledge*** *related to the subject of English. It is not as casual as traditional teaching.*  (S5) |
| --- | --- | --- | --- |
|  |  | **B) Healthy Competition** | *We used to have to wait a long time for the teacher to finish correcting our assignments and papers after exams, but with the blended learning model, we can know where we* ***went wrong in time*** *and make targeted corrections.*  (S10) |
|  |  | **C) Effective Communication** | ***Peer assessment*** *is one of my favorite classroom activities, and whenever the teacher distributes students work, the classroom can be somewhat active.*  (S4) |
|  | **Affective collection** | **A)Teacher-**  **students** | *In traditional education, teachers may be reluctant to ask students with low grades to answer questions. But in the blended learning approach, independent of our grades, we can engage in quizzes, brainstorming, topic discussions, group projects, etc. I feel that everyone* ***received adequate attention****.*  (S9) |
|  |  | **B)Students-**  **students** | *In the past, I was afraid of answering the teachers questions because I was afraid that I would make mistakes, but in this blended learning environment, I am active to challenge myself because it is really* ***encouraging from the teachers side****.*  (S12) |
|  |  | **C)Community-**  **community** | *In this blended teaching mode, everyone is more active and* ***interested especially in learning vocabulary****, and then, in traditional teaching like in junior middle school, the teacher usually follows the book and speaks in a very rigid way, and then you tend to get distracted in class.*  (S3, S4, S6) |
|  | **Communication method** | **A) Diversify tools** | *I think Chaoxing Learning app is very practical and useful, there are so many functions and these functions brought us fun activities.*  (S1) |
|  |  | **B)Convenient platform** | *I think the function of* ***submitting assignments*** *and* ***having tests*** *in Chaoxing Learning App is very good.*  (S2) |
|  |  | **C) Useful functions** | *Hope in the future, more digital tools can be introduced to our English class.*  (S8) |

**-P25**

Table 16. Themes related to social presence

| Teaching Presence | **Strategy** | **A) Using Digital Resources** | *I never knew before that there were so many rich* ***extra-curricular resources*** *for us secondary school students. In the past, when we were taught traditionally, we only listened to the teacher and if we didn’t know what to do, we put it aside, but in the blended teaching mode, the teacher provided us with many learning websites and links. if we dont understand what we know in class, we can consolidate it again and again in online classes until we learn it.*  (S12) |
| --- | --- | --- | --- |
|  |  | **B) Using Novel Tools** | *I love* ***dubbing*** *the most. I used to be very shy about speaking English, but through the* ***funny animations*** *in dubbing, Ive forgotten about being embarrassed to speak English. We can actually learn it when we speak it.*  (S6) |
|  |  | **C) Effective Instructions** | *Although the effect of cooperative learning is very good, sometimes we are not very clear about some knowledge points. It will be much better if the teacher* ***guides*** *us.*  (S7) |
|  | **Course Approach** | **A) Immense Learning** | *The role-playing in the blended learning approach allows us to* ***immerse ourselves*** *in it as if we were the protagonists.*  (S6) |
|  |  | **B)Flipped Classroom** | *This blended learning mode is helpful in overcoming students’ shyness and fear through* ***individual presentations*** *and* ***group presentations****.*  (S11) |
|  |  | **C)Learning Communities** | *The* ***learning community*** *is just like a family, we just discuss and solve the problems together. It is helpful for me to better recognize the knowledge.*  (S2) |
|  | **Meaningful Learning** | **A) Curiosity Arousal** | *In this CoI-based blended learning, the teacher would often set us up with suspense to* ***stimulate our curiosity****. I think it is very important for us to learn new knowledge.*  (S10) |
|  |  | **B) Solutions Digging** | *In the process of finding the* ***solutions to the problems****, I believe I gained knowledge.*  (S6) |
|  |  | **C) Ideas Exploration** | *When the community work together, some* ***new ideas*** *come out.*  (S4) |

**Comment 3-**Comparative Framing: Relate your findings more explicitly to previous studies such as Chen et al. (2021) and Garrison (2017). Do your results reinforce or extend their conclusions?

**Author’s revision**

**-P26-29**

The author has rewritten the discussion part. Have related the findings more explicitly to previous studies according to the two research questions. Please refer to the above mentioned text in section Results and Discussion part- comment 1, or the tracked manuscript.

**Comment 4-**Scalability: Comment on the practicality and potential scalability of the CoI-based model in other vocational or low-tech environments.

Additionally, explicitly discuss the practical significance of the blended learning intervention in terms of potential scalability in vocational settings.

**Author’s revision**

The author has rewritten the discussion part. Please refer to the above mentioned text in section Results and Discussion part- comment 1, or the tracked manuscript.

**-P26**

The findings from the first sub-research question indicated an approximately 9-point vocabulary gain with a large effect size. Chinese secondary vocational students generally start with vocabulary sizes 800-1,000 words beneath the national secondary education benchmark. Conventional vocabulary teaching methods have faced criticism for their excessive dependence on rote memorization and decontextualized word lists. Therefore, this significant enhancement is especially remarkable within the realm of Chinese secondary vocational education. The success of the VTS intervention can be systematically elucidated using the three components of the CoI framework.

...

**-P29**

What’s more, the CoI-based blended learning approach also faces challenges in low-tech vocational settings. Dindar et al.’s [132] suggestion of using fundamental mobile platforms to act as alternatives to more advanced systems can be considered. For example, in high-tech regions, AI-powered tutoring system and VR simulations can be applied; however, in low-tech areas, We-chat group and Tencent QQ group can be used instead.

- **Figures and Framework**

**Comment 1-**Annotate the framework with possible real-world actions. For example, under “Teaching Presence,” add examples like “use of recorded feedback” or “student-led microteaching.”

**Author’s revision**

-The author has added two guidelines of this framework as supplementary files to explain it.

Table 1

Guidelines for conducting the CoI-based blended learning (Steps of designing learning procedure)

| **Procedure** | **Approach** | **Descriptions** |
| --- | --- | --- |
| **Preparations** | **--** | Before classes began, students were divided into distinct communities based on their performances and personalities. Online learning forums were also created on the ChaoXing Learning Platform according to the communities. In each community, students were allocated roles such as group leader, technical recorder, designer, and connector, and were responsible for a variety of learning-related tasks. |
| **Before class** | **Online** | **Information collection:** Individual and group assignments were assigned to students, who then met and discussed using the ChaoXing Learning Platform on blended distributed terminals to share the required and collected information for the class. |
|  |  | **Personal & group tasks:** The teacher provides various learning contents through the network, students complete their individual and group assignments using various digital media, such as their smartphones and laptops, at their preferred time, space, and speed and upload them to the ChaoXing Learning Platform according to the requirements of the pre-class tasks issued by the teacher. |
|  |  | **Discussions:** Students meet and discuss to share the collected information needed for the class and upload the required documents to the ChaoXing Learning Platform. |
|  |  | **Problems proposing:** Discuss and propose the problems they were facing when doing the pre-class individual and group assignments. |
| **During class**  **(face-to-face)** | **Online + offline** | **Presentations & lectures:** Volunteers and randomly chosen students by the ChaoXing Learning Platform were asked to present their work, and teachers gave them constructive and specific feedback. |
|  |  | **Discussions:** Students in a community or inter-communities together with the teacher discuss and explore the knowledge. |
|  |  | **Summaries:** The teacher summarizes the knowledge of the present class, emphasizing teaching key points and teaching difficult points. |
|  |  | **Evaluations:** Diversification of evaluations was done by students’ self-evaluation, inter-student evaluation, teacher evaluation, and automatic evaluation by the ChaoXing Learning Platform. |
| **After class** | **Online** | **Knowledge reflecting:** Students reflect on their strengths and weaknesses in the lesson’s knowledge based on their understanding, exploration, and the teacher’s summaries. |
|  |  | **Personal & group assignments:** Consolidation and deepening of knowledge through individual and group assignments after class based on what has been learned. |
|  |  | **Knowledge integrating:** Use diagrams, mind maps, knowledge comparisons, and knowledge points to organize and integrate what have learned in class. Further knowledge application, problem analysis, and solution exploration. |
|  |  | **Problems solving:** Through students’ self-exploration, peer discussion, and teachers’ explanation, eventually lead to the resolution of the confusing questions and problems. |

Table 2

Guidelines for conducting the CoI-based blended learning (Measures should be taken during learning procedure)

| **CoI elements** | **Theme** | **Descriptions** |
| --- | --- | --- |
| **Teaching presence** | **Guidance** | **Professional instructions:** Teachers need to comprehend the theory, belief, principle, and idea underlying teaching activities or methods. They need to master the relevant professional knowledge of teaching, such as teaching environment, curriculum, teaching design, teaching mode and method, classroom management, teaching evaluation, and teaching ability of information technology. Students’ needs, qualities, differences, and motivations need to be understood as well. |
|  |  | **Timely feedback:** The feedback should be expeditious, immediate, constructive, multiple, empathetic, critical, and original. Provide timely feedback on students’ in-class participation, correctness of their performances, online and offline learning scoring, and emotions. Feedback should be made to let students be involved in the classroom, learn more about their capabilities, and maintain their self-confidence. Provide students with the direction they require to achieve their objectives. |
|  |  | **Diversify evaluations:** To ensure the authenticity, objectivity, and real-time of the entire academic evaluation, an evaluation system that combines teacher evaluation, peer evaluation, and self-evaluation; online evaluation and offline evaluation; initial evaluation and summative evaluation; dynamic evaluation and static evaluation; standard regular evaluation and random flexible evaluation should be designed. |
|  | **Emotion** | **Behavior respecting:** Teachers respond to and appreciate students’ behaviors by trusting, supporting, smiling, making eye contact, employing calming postures, having a great sense of humor, being kind, using a decent tone, and sharing positive emotions. |
|  |  | **Confidence encouraging:** Encourage, praise, and reward acceptable, learning-oriented behaviors among students. Produce positive psychological hints to boost their self-confidence, and cohesion, reduce their anxiety, and encourage them to be courageous in expressing their opinions. |
|  |  | **Interest stimulating:** Create a fun and playful environment that enhances student’s motivation and learning outcomes by appealing to their interests. Motivate and stimulate their desire to learn and seek more knowledge. Allow students to focus more on their work and eliminate monotonous lecturing routines by using authentic and interesting materials and situations. |
| **Teaching presence** | **Supervision** | **Autonomous learning:** Enhance students’ capacity for self-directed learning, including self-monitoring and self-reflection. Teachers serve as mentors to students, assisting them in identifying learning objectives, defining content and progressions, selecting methods and strategies to be used, monitoring the acquisition process, and analyzing what has been learned. Help students create realistic goals so that when they achieve them. Let students feel a sense of achievement and develop the ability to learn on their own. |
|  |  | **Self-control ability:** This includes strong self-management skills, the ability to concentrate in class, refraining from cell phone use, distraction, and gossiping with classmates. The ability to accomplish group and individual assignments assigned by the teacher self-consciously. |
|  |  | **Continuous grit:** Students own perseverance, ambition, and energy for long-term objectives, and have a clear and profound understanding motivations and goals of their actions. Be capable of regulating their emotions in response to setbacks and failures, controlling their actions, and remaining unfazed and unruffled. |
| **Social presence** | **Interaction** | **Cooperative cooperation:** Provide a safe and comfortable classroom environment and avoid using threats. Encourage students to become active, imaginative, critical, and accountable in order to achieve the team’s objective. Let students participate in the learning experience in the entirety and effective group discussion while working in groups to provide a positive experience for every participant. |
|  |  | **Healthy competition:** In a healthy competition environment, as opposed to individuals trying to outperform one another, students work to achieve organizational and common objectives. Friendship comes before competition. |
|  |  | **Effective communications:** Construct communities of blended learning among instructors and students. Create a classroom environment that is beneficial, engaging, interactive, and pleasurable. Encourage synchronous and asynchronous discussion, effective communication, and benign interaction as well as questioning, responding, and commenting among students. |
| **Social presence** | **Affective collection** | **Teacher-students:** Teachers should be able to foster an environment in which students can interact freely and safely. Relationships between teachers and students in the classroom should be characterized by respect, enjoyment, mutual trust, and comprehension. |
|  |  | **Students-students:** Positive relationships between group members to boost social interaction, individual accountability, and group competence. Learn to be tolerant and empathetic, to care for others, to praise others, to respect and accept others, and to manage conflicts between classmates. |
|  |  | **Community-community:** Strong community spirit, prompt to assist, share resources and information, and respond to inquiries. Positive and effective interactions, active learning practices, knowledge, ideas, experience, and learning products are valued and shared among communities. |
|  | **Communication method** | **Diversify tools:** Apply digital technologies, such as computer-assisted language learning (CALL) or mobile-assisted language learning (MALL), digital games, learning management systems (LMS), learning platforms, and other digital tools such as Google Form, Socrative, Kahoot!, Quizizz, Quizlet, Youdao vocabulary learning app, Wechat those have the potential to enhance teaching and learning. |
|  |  | **Convenient platform:** When implementing the CoI-based blended learning, a user-friendly platform, such as the ChaoXing Learning Platform, Rain Classroom, Ke Tang Pai, Tencent Meeting, Google Classroom, Embodo, etc., can be used. |
|  |  | **Useful functions:** Functions such as Sign-in, Information sharing, Competitive answering, Vote, Quiz, Discussion, Random candidate, Assign homework, Class report, Big data analysis, Evaluation, and E-learning design for effective interactions between teachers and students or among students. |
| **Cognitive presence** | **Strategy** | **Digital resources:** Make full use of online digital resources like Chinese University MOOC, Smart VET, Secondary Teaching Resources Website, Technical Education Website, Chongqing Cloud Platform, and resources in the ChaoXing Learning Platform. Use fundamental mobile platforms to act as alternatives in low-tech regions. |
|  |  | **Novel tools:** Using new digital teaching tools that combine text, sound, and images to enrich classroom teaching, and improve the teaching quality of teachers and the learning efficiency of students. Use fundamental mobile platforms to act as alternatives in low-tech regions. |
|  |  | **Effective instructions:** Excellent instruction should be provided. Curriculum, books, software, and other instructional materials should make sense to students. Make them engage and feel easy to apply. The objectives of each course should be specified in the instructions with varying prior knowledge. Instructions should also include new subject matter, simple language, vivid images, and examples. |
|  | **Course approach** | **Immense learning:** A form of contextualized language learning in which the learning process is situated in a diversity of virtual or actual language contexts. In addition to the interaction with the teacher making the class vivacious, active, and challenging, the students are fully engaged and aware of the learning and processes taking place throughout the entire class period. |
|  |  | **Flipped classroom:** Classes are held in digital environments that promote the development of students’ competencies through cooperative learning, problem-solving, and critical thinking. An emphasis is additionally put on dynamic interaction and the transfer of knowledge using digital tools in a collaborative and idea-sharing spirit. Students learn various topics separately utilizing video tutorials before applying their knowledge in class by working on practical exercises. Students are encouraged to learn alone and in groups, which facilitates the exchange of learning content throughout communities and, as a result, improves students’ problem-solving abilities. |
|  |  | **Learning communities:** Students allocate to small groups to master academic content. Classroom discussions, presentations, and group projects are progressively transformed into collaborative learning through the use of technology. |
|  | **Meaningful learning** | **Curiosities arousal:** Piquing students’ motivation and curiosity about the material to be taught. For example, use surprising demonstrations, the comparison of subjects to students’ own lives, or allow students to find out information by themselves. |
|  |  | **Solutions digging:** Encourage students to establish and reinforce meaning by having ongoing discussions and reflections. Encouraging critical thinking in a community of students through emotional, interactive, and coherent responses to help reach cognitive learning objectives. |
|  |  | **Ideas exploration:** Sharing the knowledge and information gained through group discussions. Encourage critical thinking, problem-solving, and new ideas-creating abilities among students. |

**Comment 2-**Offer low-tech adaptations for under-resourced settings.

**Author’s revision**

-The author has added a guideline of this framework as a supplementary file to explain it. See the above provided guidelines in the two tables.

-**Digital resources:** Make full use of online digital resources like Chinese University MOOC, Smart VET, Secondary Teaching Resources Website, Technical Education Website, Chongqing Cloud Platform, and resources in the ChaoXing Learning Platform. Use fundamental mobile platforms to act as alternatives in low-tech regions.

**-Novel tools:** Using new digital teaching tools that combine text, sound, and images to enrich classroom teaching, and improve the teaching quality of teachers and the learning efficiency of students. Use fundamental mobile platforms to act as alternatives in low-tech regions.

- **Language and Formatting**

**Comments-**
- Grammar and phrasing require minor editing. For instance:
- “The lacking amount of vocabulary…” → “The lack of vocabulary…”
- “Their VTS and VLM…” → Use full terms on first use in each section.

-The Vocabulary Test compromises 40 multiple-choice questions…

-The Vocabulary Test consists of 40 multiple-choice questions
- Ensure verb tenses are consistent, particularly in the methodology and results sections.
- APA/PLOS formatting for p-values and statistical terms should be checked.

• Tables and figures should be uniformly formatted. Ensure that table captions are clear and that all abbreviations (e.g., VTS, VLM) are defined in each table

In the literature review, ensure that all in-text citations are consistent and correctly formatted.

**Author’s revision**

>The author has checked the grammar throughout the manuscript. See the manuscript.

>**P1-**“The mechanical traditional way of teaching and their lack of vocabulary toward the target language often demotivate them in learning”.

>Full terms like VTS, VLM, CoI, TP, SP, CP has been used on the first mention in each section. See the tracked manuscript.

>**P8-**“In this study, a researcher-developed Vocabulary Test was employed for both the pre-test and post-test. The test consisted of 40 multiple-choice questions, each valued at one point, yielding a maximum score of 40”.

>The author has checked the verb tenses are consistent, particularly in the methodology and results sections. See the manuscript.

>The author has revised p-values and statistical terms. See the tracked manuscript.

>Tables and figures has been uniformly formatted. All abbreviations (e.g., VTS, VLM) are defined in each table. See the tracked manuscript.

>The author has checked the in-text citations in the literature review part. See the tracked manuscript.

- **Limitations and Future Research**

**Comment 1-**Propose longitudinal studies on vocabulary retention and motivation.

**Author’s revision**

**-P31**

“Second, due to time constraints, this study was designed as a quasi-experiment with a duration of 13 weeks, to enhance analytical rigor, future research should incorporate longitudinal methodologies to explore causal relationships more robustly and validate findings on vocabulary retention and motivation. ”

**Comment 2-**Suggest cross-discipline comparisons or cross-platform CoI applications.

**Author’s revision**

**-P30**

“First, according to the characteristics of vocational majors, a limitation in terms of the gender imbalance among students exists in this study. Future studies could purposefully select gender-balanced cohorts from a variety of vocational disciplines to examine the effectiveness of the CoI framework in order to address these discrepancies. ”

**Comment 3-**Discuss gender-based or socio-economic implications in vocational settings.

**Author’s revision**

**-P30**

“Although the sample’s gender imbalance (81.8% male) reflects the demographic reality of Chinese vocational schools, it restricts the possibility to assess the potential influences of gender differences on CoI-based blended learning. Therefore, research on the effectiveness of this approach should focus on socioeconomic or gender-based demographics.”

**Reviewer #1 comments**

- **Figures & Framework (Fig 5.1)**

**Comment 1-**I would like to suggest if figure 5.1 can be re designed or replace as it seems little unclear at present.

**Author’s revision**

-The author has added two guidelines of this framework as a supplementary file to explain it.

Table 1

Guidelines for conducting the CoI-based blended learning (Steps of designing learning procedure)

| **Procedure** | **Approach** | **Descriptions** |
| --- | --- | --- |
| **Preparations** | **--** | Before classes began, students were divided into distinct communities based on their performances and personalities. Online learning forums were also created on the ChaoXing Learning Platform according to the communities. In each community, students were allocated roles such as group leader, technical recorder, designer, and connector, and were responsible for a variety of learning-related tasks. |
| **Before class** | **Online** | **Information collection:** Individual and group assignments were assigned to students, who then met and discussed using the ChaoXing Learning Platform on blended distributed terminals to share the required and collected information for the class. |
|  |  | **Personal & group tasks:** The teacher provides various learning contents through the network, students complete their individual and group assignments using various digital media, such as their smartphones and laptops, at their preferred time, space, and speed and upload them to the ChaoXing Learning Platform according to the requirements of the pre-class tasks issued by the teacher. |
|  |  | **Discussions:** Students meet and discuss to share the collected information needed for the class and upload the required documents to the ChaoXing Learning Platform. |
|  |  | **Problems proposing:** Discuss and propose the problems they were facing when doing the pre-class individual and group assignments. |
| **During class**  **(face-to-face)** | **Online + offline** | **Presentations & lectures:** Volunteers and randomly chosen students by the ChaoXing Learning Platform were asked to present their work, and teachers gave them constructive and specific feedback. |
|  |  | **Discussions:** Students in a community or inter-communities together with the teacher discuss and explore the knowledge. |
|  |  | **Summaries:** The teacher summarizes the knowledge of the present class, emphasizing teaching key points and teaching difficult points. |
|  |  | **Evaluations:** Diversification of evaluations was done by students’ self-evaluation, inter-student evaluation, teacher evaluation, and automatic evaluation by the ChaoXing Learning Platform. |
| **After class** | **Online** | **Knowledge reflecting:** Students reflect on their strengths and weaknesses in the lesson’s knowledge based on their understanding, exploration, and the teacher’s summaries. |
|  |  | **Personal & group assignments:** Consolidation and deepening of knowledge through individual and group assignments after class based on what has been learned. |
|  |  | **Knowledge integrating:** Use diagrams, mind maps, knowledge comparisons, and knowledge points to organize and integrate what have learned in class. Further knowledge application, problem analysis, and solution exploration. |
|  |  | **Problems solving:** Through students’ self-exploration, peer discussion, and teachers’ explanation, eventually lead to the resolution of the confusing questions and problems. |

Table 2

Guidelines for conducting the CoI-based blended learning (Measures should be taken during learning procedure)

| **CoI elements** | **Theme** | **Descriptions** |
| --- | --- | --- |
| **Teaching presence** | **Guidance** | **Professional instructions:** Teachers need to comprehend the theory, belief, principle, and idea underlying teaching activities or methods. They need to master the relevant professional knowledge of teaching, such as teaching environment, curriculum, teaching design, teaching mode and method, classroom management, teaching evaluation, and teaching ability of information technology. Students’ needs, qualities, differences, and motivations need to be understood as well. |
|  |  | **Timely feedback:** The feedback should be expeditious, immediate, constructive, multiple, empathetic, critical, and original. Provide timely feedback on students’ in-class participation, correctness of their performances, online and offline learning scoring, and emotions. Feedback should be made to let students be involved in the classroom, learn more about their capabilities, and maintain their self-confidence. Provide students with the direction they require to achieve their objectives. |
|  |  | **Diversify evaluations:** To ensure the authenticity, objectivity, and real-time of the entire academic evaluation, an evaluation system that combines teacher evaluation, peer evaluation, and self-evaluation; online evaluation and offline evaluation; initial evaluation and summative evaluation; dynamic evaluation and static evaluation; standard regular evaluation and random flexible evaluation should be designed. |
|  | **Emotion** | **Behavior respecting:** Teachers respond to and appreciate students’ behaviors by trusting, supporting, smiling, making eye contact, employing calming postures, having a great sense of humor, being kind, using a decent tone, and sharing positive emotions. |
|  |  | **Confidence encouraging:** Encourage, praise, and reward acceptable, learning-oriented behaviors among students. Produce positive psychological hints to boost their self-confidence, and cohesion, reduce their anxiety, and encourage them to be courageous in expressing their opinions. |
|  |  | **Interest stimulating:** Create a fun and playful environment that enhances student’s motivation and learning outcomes by appealing to their interests. Motivate and stimulate their desire to learn and seek more knowledge. Allow students to focus more on their work and eliminate monotonous lecturing routines by using authentic and interesting materials and situations. |
| **Teaching presence** | **Supervision** | **Autonomous learning:** Enhance students’ capacity for self-directed learning, including self-monitoring and self-reflection. Teachers serve as mentors to students, assisting them in identifying learning objectives, defining content and progressions, selecting methods and strategies to be used, monitoring the acquisition process, and analyzing what has been learned. Help students create realistic goals so that when they achieve them. Let students feel a sense of achievement and develop the ability to learn on their own. |
|  |  | **Self-control ability:** This includes strong self-management skills, the ability to concentrate in class, refraining from cell phone use, distraction, and gossiping with classmates. The ability to accomplish group and individual assignments assigned by the teacher self-consciously. |
|  |  | **Continuous grit:** Students own perseverance, ambition, and energy for long-term objectives, and have a clear and profound understanding motivations and goals of their actions. Be capable of regulating their emotions in response to setbacks and failures, controlling their actions, and remaining unfazed and unruffled. |
| **Social presence** | **Interaction** | **Cooperative cooperation:** Provide a safe and comfortable classroom environment and avoid using threats. Encourage students to become active, imaginative, critical, and accountable in order to achieve the team’s objective. Let students participate in the learning experience in the entirety and effective group discussion while working in groups to provide a positive experience for every participant. |
|  |  | **Healthy competition:** In a healthy competition environment, as opposed to individuals trying to outperform one another, students work to achieve organizational and common objectives. Friendship comes before competition. |
|  |  | **Effective communications:** Construct communities of blended learning among instructors and students. Create a classroom environment that is beneficial, engaging, interactive, and pleasurable. Encourage synchronous and asynchronous discussion, effective communication, and benign interaction as well as questioning, responding, and commenting among students. |
| **Social presence** | **Affective collection** | **Teacher-students:** Teachers should be able to foster an environment in which students can interact freely and safely. Relationships between teachers and students in the classroom should be characterized by respect, enjoyment, mutual trust, and comprehension. |
|  |  | **Students-students:** Positive relationships between group members to boost social interaction, individual accountability, and group competence. Learn to be tolerant and empathetic, to care for others, to praise others, to respect and accept others, and to manage conflicts between classmates. |
|  |  | **Community-community:** Strong community spirit, prompt to assist, share resources and information, and respond to inquiries. Positive and effective interactions, active learning practices, knowledge, ideas, experience, and learning products are valued and shared among communities. |
|  | **Communication method** | **Diversify tools:** Apply digital technologies, such as computer-assisted language learning (CALL) or mobile-assisted language learning (MALL), digital games, learning management systems (LMS), learning platforms, and other digital tools such as Google Form, Socrative, Kahoot!, Quizizz, Quizlet, Youdao vocabulary learning app, Wechat those have the potential to enhance teaching and learning. |
|  |  | **Convenient platform:** When implementing the CoI-based blended learning, a user-friendly platform, such as the ChaoXing Learning Platform, Rain Classroom, Ke Tang Pai, Tencent Meeting, Google Classroom, Embodo, etc., can be used. |
|  |  | **Useful functions:** Functions such as Sign-in, Information sharing, Competitive answering, Vote, Quiz, Discussion, Random candidate, Assign homework, Class report, Big data analysis, Evaluation, and E-learning design for effective interactions between teachers and students or among students. |
| **Cognitive presence** | **Strategy** | **Digital resources:** Make full use of online digital resources like Chinese University MOOC, Smart VET, Secondary Teaching Resources Website, Technical Education Website, Chongqing Cloud Platform, and resources in the ChaoXing Learning Platform. Use fundamental mobile platforms to act as alternatives in low-tech regions. |
|  |  | **Novel tools:** Using new digital teaching tools that combine text, sound, and images to enrich classroom teaching, and improve the teaching quality of teachers and the learning efficiency of students. Use fundamental mobile platforms to act as alternatives in low-tech regions. |
|  |  | **Effective instructions:** Excellent instruction should be provided. Curriculum, books, software, and other instructional materials should make sense to students. Make them engage and feel easy to apply. The objectives of each course should be specified in the instructions with varying prior knowledge. Instructions should also include new subject matter, simple language, vivid images, and examples. |
|  | **Course approach** | **Immense learning:** A form of contextualized language learning in which the learning process is situated in a diversity of virtual or actual language contexts. In addition to the interaction with the teacher making the class vivacious, active, and challenging, the students are fully engaged and aware of the learning and processes taking place throughout the entire class period. |
|  |  | **Flipped classroom:** Classes are held in digital environments that promote the development of students’ competencies through cooperative learning, problem-solving, and critical thinking. An emphasis is additionally put on dynamic interaction and the transfer of knowledge using digital tools in a collaborative and idea-sharing spirit. Students learn various topics separately utilizing video tutorials before applying their knowledge in class by working on practical exercises. Students are encouraged to learn alone and in groups, which facilitates the exchange of learning content throughout communities and, as a result, improves students’ problem-solving abilities. |
|  |  | **Learning communities:** Students allocate to small groups to master academic content. Classroom discussions, presentations, and group projects are progressively transformed into collaborative learning through the use of technology. |
|  | **Meaningful learning** | **Curiosities arousal:** Piquing students’ motivation and curiosity about the material to be taught. For example, use surprising demonstrations, the comparison of subjects to students’ own lives, or allow students to find out information by themselves. |
|  |  | **Solutions digging:** Encourage students to establish and reinforce meaning by having ongoing discussions and reflections. Encouraging critical thinking in a community of students through emotional, interactive, and coherent responses to help reach cognitive learning objectives. |
|  |  | **Ideas exploration:** Sharing the knowledge and information gained through group discussions. Encourage critical thinking, problem-solving, and new ideas-creating abilities among students. |

**Reviewer #2 comments**

- **Literature Review – Theoretical Integration and Relevance**

**Comment 1-Integration of Digital Transformation Perspectives:** Although the review discusses blended learning and CoI, it would benefit from more discussion of the digital transformation aspect. For example, your work:
Al Fraidan, A. & Alaliwi, M. (2024). Digital Transformation for Sustainable English Language Learning: Insights from Saudi Arabia and Global Perspectives
could be cited to provide a cross-cultural or global perspective on how digital tools enhance language learning, thereby reinforcing the rationale for using a technology-based platform like Chaoxing Learning App.

**Author’s revision**

**-P2**

“In recent years, the adoption of blended learning has surged, owing to its flexibility and the integration of digital tools that foster both independent and collaborative learning [1],[5],[6]. ”

**-P7**

“Digital tools provide English language learners the chance to practice their language skills continuously, receive immediate feedback, and assess in real time, all of which are essential for improving their language retention and skill set. ”

**-P7**

“In order to monitor students’ progress in real time, several nations have long integrated digital tools including Google Classroom, Edmodo, Socrative, Classting, Madrasati, Rosetta Stone, and Busuu. Teachers can use these tools to produce automatically graded projects and quizzes that give instant feedback.”

**Comment 2-**Motivation and Test Anxiety in Language Learning:
• Al Fraidan, A. & AlDossari, M. S. A. (2025). Navigating the Lexical Labyrinth: Vocabulary Test Anxiety, Teacher Strictness, and Strategic Mastery in ESL Assessment.
This study provides insights into the affective components of language learning which could complement your discussion of vocabulary learning motivation.

**Author’s revision**

**-P29**

“In the same way, Past studies [129-131] all noted that structured and supportive learning environments contribute to better academic resilience. Teachers ought to provide guiding roles by offering helpful feedback, impartial observations, and constructive views during tasks. ”

**Comment 3-Vocabulary Assessment and Motivation:** The review would also be strengthened by linking vocabulary testing and motivation with recent findings. You might include:
• Al Fraidan, A. (2025). Evaluating Lexical Competency in Saudi Arabia’s Hybridized EFL Ecosystem: A Taxonomic Exploration of Vocabulary Assessment Modalities and the Imperative for AI-Enhanced Adaptive Testing
to support the discussion of vocabulary assessment challenges and the need for innovative instructional designs.

**Author’s revision**

**-P6**

“Recent study [73] underscores the need for adaptive and interactive assessment modalities to enhance both vocabulary acquisition and learner motivation. ”

**-P29**

“To address this problem, [73] suggested that the use of AI-driven platforms can facilitate more tailored and adaptive assessment experiences that correspond with the requirements of a contemporary, blended educational framework.”

**Comment 4-Excerpts for Revision:**
For instance, the sentence:
“Students were frequently provided with voluminous paper-based vocabulary books and subjected to the laborious process of passive learning.”
could be revised for clarity and depth to:
“Traditionally, students have relied on extensive paper-based vocabulary exercises, a method that often reduces engagement and limits the opportunity for contextualized learning. Recent studies (e.g., Al Fraidan, 2025) underscore the need for adaptive and interactive assessment modalities to enhance both vocabulary acquisition and learner motivation.”

**Author’s revision**

**-P6**

“Students have to rely on extensive paper-based vocabulary exercises, a method that often reduces engagement and limits contextualized learning opportunities. Recent study [73] underscores the need for adaptive and interactive assessment modalities to enhance both vocabulary acquisition and learner motivation.”

- **Methodology – Design, Intervention, and Data Analysis**

**Comment 1-Design and Sampling:**
**• Clarification Needed:**
While the use of a quasi-experimental design is appropriate, further detail is needed on the sampling and group assignment process. Explain how you ensured the experimental and control groups were comparable in terms of English proficiency and demographic factors. Specify whether random assignment (or matching) was used.
**• Suggestion:**
Include a brief statement on how you minimized potential confounders. For example:
“Using purposive sampling, both groups were matched on baseline vocabulary scores and demographic characteristics, and pre-test homogeneity was confirmed via an independent samples t-test (t(84)=0.20, p=0.83).”

**Author’s revision**

**-P8**

“Using purposive sampling, both groups were matched on baseline vocabulary scores and demographic characteristics, and pre-test homogeneity was confirmed via an independent samples t-test (t(84)=0.20, p=0.83).”

**Comment 2-Intervention Description:
• Enhancement of Details:**
The intervention plan is described, but the link between CoI elements and specific blended learning activities can be clearer. For instance, the activities in Table 1 (elements, categories, and activities) should be explicitly tied back to the theoretical constructs of TP, SP, and CP.
**• Suggestion:**
Provide a flowchart or timeline that maps each intervention phase to the corresponding CoI element(s). Clarify how online discussions, quizzes, and group tasks contribute to cognitive and social presence.

**Author’s revision**

**-P13**

The author has added Table 2. The Application CoI Framework- Take lesson lets go shopping as an example in the manuscript.

| **Teaching Steps** | **Teachers Activities** | **CoI Application** | **Students Activities** | **CoI Application** |
| --- | --- | --- | --- | --- |
| **Before- Class** | **Preview (Online)**  1.Create an online course and groups with the ChaoXing Learning App, and instruct students to log in using a QR code. | **Teaching Presence（TP）-** Design & Organization | **Preview (Online)**  1.Sign in the class and belonged group according to the invitation QR code. | -- |
|  | **Preview (Online)**  2.Release a short video -“shopping” to students on the ChaoXing Learning App:  <https://m.v.qq.com/play.html?vid=h07651mgg1c&cid=,> and ask them to finish preview task card 1 by group. | **Teaching Presence（TP）-** Design & Organization; Facilitating Discourse  **Social Presence（SP) -**  Affective Expression; Group Cohesion | **Preview (Online)**  2.Watch the short video- “shopping” and finish preview task card 1 by group. | **Cognitive Presence (CP)-** Triggering Event  **Social Presence（SP) -**  Affective Expression; Group Cohesion |
|  | **Preview (Online)**  3.Instruct students to read and preview the short reading in Exercise A on page P21, the important phrases in Exercise F on page P22, and the ten key words on page P164, and to complete preview task card 2 individually. | **Teaching Presence（TP）-** Design & Organization | **Preview (Online)**  3.Complete the preview tasks of the short reading in Exercise A on page P21, Key sentences in Exercise F on page P22, and 10 key words on page P164 and finish preview task card 2 personally. | **Cognitive Presence (CP)-** Triggering Event |
|  | **Preview (Online)**  4.Instruct students to research Martins Department Store, 24-7 Convenience Store, Sams Food Market, Hero Books, Shoe Emporium, Dress for Less, and Thrift Central, and complete preview task card 3 collaboratively. | **Teaching Presence（TP）-** Design & Organization; Facilitating Discourse  **Social Presence（SP) -**  Affective Expression; Open Communication; Group Cohesion | **Preview (Online)**  4.Investigate details regarding Martins Department Store, 24-7 Convenience Store, Sams Food Market, Hero Books, Shoe Emporium, Dress for Less, and Thrift Central, then complete the preview task card 3 collaboratively as a group. | **Cognitive Presence (CP)-** Triggering Event  **Social Presence（SP) -**  Affective Expression; Open Communication;  Group Cohesion |
|  | **Preview (Online)**  5.Release a micro lesson on the use of lexical verbs in the simple present tense: https://b23.tv/0oOhiL6 Instruct students on the ChaoXing Learning App to collaboratively complete preview task card 4. | **Teaching Presence（TP）-** Design & Organization; Facilitating Discourse  **Social Presence（SP) -**  Affective Expression; Open Communication; Group Cohesion | **Preview (Online)**  5.Watch the micro lesson of lexical verb used in Simple present tense: <https://b23.tv/0oOhiL6,> and finish preview task card 4 by group. | **Cognitive Presence (CP)-** Triggering Event  **Social Presence（SP) -**  Affective Expression; Open Communication; Group Cohesion |
|  | **Preview (Online)**  6.Ask students to upload preview tasks cards to the ChaoXing Learning App and do the self-assessment of preview tasks. | **Teaching Presence（TP）-** Design & Organization | **Preview (Online)**  6.Finish preview task cards and upload them to the ChaoXing Learning App and do the self-assessment of preview tasks. | -- |
| **In-Class** | **Lead-in (Online+Face-to-face)**  1.Issues a sign-in gesture on the ChaoXing Learning App. **(1min)** | **Teaching Presence（TP）-** Design & Organization | **Lead-in (Online+Face-to-face)**  1. Sign-in through the ChaoXing Learning App by gesture. | -- |
|  | **Lead-in (Online+Face-to-face)**  2.Present students with images related to shopping and pose the question: Do you enjoy shopping? Request students to generate and list the vocabulary related to shopping on the screen. Present the subject of the current lesson.  **(2min)** | **Teaching Presence（TP）-** Design & Organization; Facilitating Discourse  **Social Presence（SP) -**  Affective Expression; Open Communication | **Lead-in (Online+Face-to-face)**  2.Respond to the instructor’s inquiries. Generating and listing the vocabulary related to shopping that they are familiar with on the screen. | **Cognitive Presence (CP)-** Triggering Event  **Social Presence（SP) -**  Affective Expression; Open Communication |
|  | **Vocabulary Learning (Online+Face-to-face)**  3.Read about Van on page 21 with students and introduce Van’s problem to students. **(2min)** | **Teaching Presence（TP）-** Design & Organization; Facilitating Discourse  **Social Presence（SP) -**  Affective Expression; Open Communication | **Vocabulary Learning (Online+Face-to-face)**  3.Read about Van on page 21with the teacher and get to know Van’s problem | **Cognitive Presence (CP)-** Triggering Event  **Social Presence（SP) -**  Affective Expression; Open Communication |
|  | **Vocabulary Learning (Online+Face-to-face)**  4.Explain 10 key words and 1 sentence pattern of this lesson. **(8min)** | **Teaching Presence（TP）-** Design & Organization; Facilitating Discourse; Direct Instruction  **Social Presence（SP) -**  Affective Expression; Open Communication. | **Vocabulary Learning (Online+Face-to-face)**  4.Learn 10 key words and 1 sentence pattern of this lesson. | **Cognitive Presence (CP)-** Exploration  **Social Presence（SP) -**  Affective Expression;  Open Communication. |
|  | **Vocabulary Learning (Online+Face-to-face)**  5.Release the Seewo words game through the ChaoXing Learning App and announce the rules to the students. **(8min)** | **Teaching Presence（TP）-** Design & Organization; Facilitating Discourse  **Social Presence（SP)-**  Open Communication; Group Cohesion | **Vocabulary Learning (Online+Face-to-face)**  5.Each group selects one representative to compete in pairs, with the victor remaining until the conclusion of the game. | **Cognitive Presence (CP)-** Exploration  **Social Presence（SP)-**  Open Communication;  Group Cohesion |
|  | **Vocabulary Learning**  **(Online+Face-to-face)**  6.Select students at random to present the information they gathered on various stores before class.  **(6min)** | **Teaching Presence（TP）-** Design & Organization; Facilitating Discourse  **Social Presence（SP) -**  Affective Expression; Open Communication; Group Cohesion | **Vocabulary Learning (Online+Face-to-face)**  6.Present the information about different stores that they collected before class by group. | **Cognitive Presence (CP)-** Exploration  **Social Presence（SP) -**  Affective Expression;  Open Communication; Group Cohesion |
|  | **Vocabulary Learning (Online+Face-to-face)**  7.Ask students to help Van find the right stores to buy her needed items and finish Exercise B & C. **(5min)** | **Teaching Presence（TP）-** Design & Organization; Facilitating Discourse; Direct Instruction  **Social Presence（SP) -**  Affective Expression; Open Communication | **Vocabulary Learning (Online+Face-to-face)**  7.Help Van find the right stores to buy her needed items and finish Exercise B & C. | **Cognitive Presence (CP)-** Exploration  **Social Presence（SP) -**  Affective Expression;  Open Communication |
|  | **Vocabulary Learning (Online+Face-to-face)**  8.Provide directives for students to conduct Exercise D: Listen to Van and her spouse. Identify and circle the best location to get each item.  **(3min)** | **Teaching Presence（TP）-** Design & Organization; Facilitating Discourse; Direct Instruction  **Social Presence（SP) -**  Affective Expression; Open Communication. | **Vocabulary Learning (Online+Face-to-face)**  8.Complete Exercise D: Listen to Van and her husband. Circle the best place to get each item. | **Cognitive Presence (CP)-** Exploration  **Social Presence（SP) -**  Affective Expression;  Open Communication. |
|  | **Conversation Practicing (Online+Face-to-face)**  9.Instruct students to utilize the Youdao Learning App to capture images of the sentence pattern from Exercise F on page 22 and subsequently read aloud using the App.  **(5min)** | **Teaching Presence（TP）-** Design & Organization; Facilitating Discourse | **Conversation Practicing (Online+Face-to-face)**  9.Utilize the Youdao Learning App to capture images of the phrase structure from Exercise F on page 22 and subsequently read them using the App. | **Cognitive Presence (CP)-**  Exploration |
|  | **Conversation Practicing (Online+Face-to-face)**  10.Students are randomly chosen via the “shake” function of the ChaoXing Learning App to construct phrases incorporating “shoes”, “shirts”, “dictionary”, “bread”, “cheese”, and “fruit” within new dialogues utilizing the acquired sentence structure.  **(7min)** | **Teaching Presence（TP）-** Design & Organization; Facilitating Discourse  **Social Presence（SP) -**  Affective Expression; Open Communication | **Conversation Practicing (Online+Face-to-face)**  10.The chosen students construct phrases using “shoes,” “shirts,” “dictionary,” “bread,” “cheese,” and “fruit” within new dialogues utilizing the learnt sentence structure. | **Cognitive Presence (CP)-**  Exploration  **Social Presence（SP) -**  Affective Expression;  Open Communication |
|  | **Grammar Tips**  **(Online+Face-to-face)**  11.Facilitate a theme discourse on the ChaoXing Learning App and instruct students to analyze, deliberate, and synthesize the use of the term “shop” in various phrases within Exercise G: Examine the chart alongside your classmates and instructor.  **(5min)** | **Teaching Presence（TP）-** Design & Organization; Facilitating Discourse  **Social Presence（SP) -**  Affective Expression; Open Communication | **Grammar Tips**  **(Online+Face-to-face)**  11.Refer to Exercise G: Engage in a debate regarding the teacher’s inquiry and submit your response on the ChaoXing Learning App via theme discourse. | **Cognitive Presence (CP)-**  Exploration  **Social Presence（SP) -**  Affective Expression;  Open Communication |
|  | **Grammar Tips**  **(Online+Face-to-face)**  12.Provide more instances of lexical verbs utilized in the simple present tense and instruct students to summarize and conclude. Exercise H: Fill in the sentences with the appropriate form of the verb “shop”.  **(6min)** | **Teaching Presence（TP）-** Design & Organization; Direct Instruction | **Grammar Tips**  **(Online+Face-to-face)**  12.Summarize the application of lexical verbs in the simple present tense and conclude. Exercise H: Fill in the sentences with the appropriate form of the verb “shop”. | **Cognitive Presence (CP)-**  Exploration |
|  | **Summary**  **(Online+Face-to-face)**  13.Distribute an online survey via the ChaoXing Learning App and request students to create a bar graph to analyze the frequency of classmates purchasing at various types of Chinese retailers by group.  **(8min)** | **Teaching Presence（TP）-** Design & Organization; Facilitating Discourse  **Social Presence（SP) -**  Affective Expression; Open Communication; Group Cohesion | **Summary**  **(Online+Face-to-face)**  13.Complete the online survey and create a bar graph to analyze the frequency of classmates shopping at various types of Chinese retailers by group. | **Cognitive Presence (CP)-**  Integration  **Social Presence（SP) -**  Affective Expression;  Open Communication;  Group Cohesion |
|  | **Summary**  **(Online+Face-to-face)**  14.Review, summarize and fill in the mind map with knowledge learned in this lesson together with students. **(7min)** | **Teaching Presence（TP）-** Design & Organization; Facilitating Discourse; Direct Instruction  **Social Presence（SP) -**  Affective Expression; Open Communication | **Summary**  **(Online+Face-to-face)**  14.Review, summarize and fill in the mind map with knowledge learned in this lesson together with the teacher. | **Cognitive Presence (CP)-**  Integration  **Social Presence（SP) -**  Affective Expression;  Open Communication |
|  | **Evaluation**  **(Online+Face-to-face)**  15.Facilitate students in completing the self-assessment form, peer evaluation form, and teacher evaluation form via the ChaoXing Learning App.  **(5min)** | **Teaching Presence（TP）-** Design & Organization; Facilitating Discourse; Direct Instruction  **Social Presence（SP) -**  Affective Expression; Open Communication; Group Cohesion | **Evaluation**  **(Online+Face-to-face)**  15.Complete the students self-evaluation form, peer evaluation form through the ChaoXing Learning App. | **Cognitive Presence (CP)-**  Integration  **Social Presence（SP) -**  Affective Expression;  Open Communication;  Group Cohesion |
| **After- class** | **Expanding (Online)**  1.Discharge Active Task. Visit a shopping mall or utilize the Internet. Identify the names of three apparel retailers that you favor. Prepare a report and post it to the ChaoXing Learning App as a group. | **Teaching Presence（TP）-** Design & Organization; Facilitating Discourse; Affective Expression  **Social Presence（SP)-**  Open Communication; Group Cohesion | **Expanding**  **(Online)**  1.Execute Active Task. Visit a shopping mall or utilize the Internet. Identify the names of three apparel retailers that you favor. Prepare a report and post it to the ChaoXing Learning App as a group. | **Cognitive Presence (CP)-**  Resolution  **Social Presence（SP) -**  Affective Expression;  Open Communication;  Group Cohesion |
|  | **Expanding (Online)**  2.Instruct students to complete pertinent exercises in their exercise books and upload photographs of them to the ChaoXing Learning App. | **Teaching Presence（TP）-** Design & Organization | **Expanding**  **(Online)**  2.Finish relevant exercises on exercise book and upload it to the ChaoXing Learning App by photos. | **Cognitive Presence (CP)-**  Resolution |
|  | **Expanding (Online)**  3.Ask students to finish vocabulary exercise on the ChaoXing Learning App. | **Teaching Presence（TP）-** Design & Organization | **Expanding**  **(Online)**  3.Finish vocabulary exercise on the ChaoXing Learning App. | **Cognitive Presence (CP)-**  Resolution |
|  | **Evaluation (Online)**  4.Assess students’ homework and prompt them to conduct self-evaluations of their extracurricular assignments. | **Teaching Presence（TP）-** Design & Organization | **Evaluation (Online)**  4.Do self-assessment about their after-class tasks. | **--** |

- **Data Collection and Instrumentation:**

**Comment 1-Instrumentation Validity:**
The researcher-developed vocabulary test and VLM questionnaire are mentioned, and Cronbach’s Alpha values are provided. However, additional details on the validation process (e.g., expert review, pilot testing) would strengthen the methodological rigor.

**Author’s revision**

**-P8-9**

The author has added the Instruments section to describe the reliability and Validity of the instruments of this study.

**Instruments**

**Vocabulary pre-test and post-test**

Multiple-choice assessment is the most often employed evaluations to measure the receptive vocabulary knowledge of EFL/ESL learners [93]. In this study, a researcher-developed Vocabulary Test was employed for both the pre-test and post-test. The test consisted of 40 multiple-choice questions, each valued at one point, yielding a maximum score of 40. Students were mandated to select one accurate response from the four alternatives: A, B, C, or D. The Vocabulary Test questions were subsequently prepared in accordance with the national standard English textbook for secondary vocational students: New Model English 1 (2nd edition). The items were established in accordance with the standard Classified College Entrance Examination (CCEE) test package. Following the creation of the draft, it was subjected to evaluation and approval by a panel of two experts in Chinese secondary vocational EFL education. The Cronbach’s Alpha for the Vocabulary Test was 0.718, indicating an acceptable level [94].

Prior to the CoI-based blended learning intervention, a pre-test was administered using the aforementioned researcher-developed Vocabulary Test to assess the pre-existing Vocabulary Test Scores (VTS) of the two target groups. The objective was to evaluate the students’ initial proficiency and set a baseline for comparison in the post-test. The post-test is a standardized evaluation administered at the conclusion of the intervention. It seeks to evaluate students attainment of the particular intervention [95]. Naps et al.[96] asserted that care must be given to ensure that the pre-test and post-test are isomorphic. This is most effectively achieved by employing the same questions in a varied sequence. Consequently, the same Vocabulary Test utilized in the pre-test was administered in the post-test, with the items randomized and reorganized to prevent students from depending on rote memorization. To maintain validity, the students were not informed of the post-test, preventing them from recalling the words or questions from the pre-test [97].

**Vocabulary learning motivation questionnaire**

The VLM questionnaire consisted of two sections. The first section aimed to gather demographic information from students, including gender and fundamental details regarding their English language acquisition. The second section concentrated on the VLM inquiries. This questionnaire was adapted from [57], who cross-culturally modified the Vocabulary Learning Motivation Questionnaire [98] and validated it within a Chinese high school context. Initially comprising 20 items, it was subsequently refined to 19 items following the two aforementioned experts evaluation due to redundancy in the descriptions of two questions. Students were instructed to respond to all inquiries within each section using a five-point Likert-type scale, ranging from “strongly agree” (five points) to “strongly disagree” (one point). The Cronbach’s Alpha for the VLM questionnaire indicated a reliability of 0.855, categorized as a Good level [94]. Prior to the CoI-based blended learning intervention, the pre-intervention was conducted among the students in both groups using this VLM questionnaire. In the post-intervention phase, the same VLM questionnaire was administered but with the items rearranged and reordered.

**Semi-structured interview protocol**

After the CoI-based blended learning intervention, semi-structured interviews were performed with 12 students from the experimental group. The interviews included 15 open-ended questions, and participation was voluntary. The objective was to investigate students’ perspectives of the CoI-based blended learning approach and to elucidate the quantitative findings further. Initially, 10 open-ended questions were formulated. Given the traits of secondary vocational school students, who typically exhibit reticence, two local experts proposed a final version of 15 open-ended questions.

**Data collecting and analysis**

In the first phase, the researcher obtained ethical approval from the Technician College. Prior to the study, all participants received comprehensive information regarding the objectives and methodologies of this study. They were obligated to provide written consent forms for participation. Prior to the main study, a pilot study was conducted to obtain feedback regarding the reliability of the instruments [99].

...

**Comment 2-Qualitative Data:**
The description of the thematic analysis (using Open, Axial, and Selective Coding) is clear. Yet, consider providing examples of the coding process and how inter-coder reliability was established.

**Author’s revision**

**-P11**

Table 1. Coding example of interview transcripts

According to [101], to achieve trustworthiness, selecting individuals with diverse backgrounds enhances the probability of providing insight into the study subject from multiple perspectives. Therefore, the interviewees were students of four high proficiency level, four medium proficiency level, and four low proficiency level students according to their Vocabulary Test scores. Initially, written transcripts were generated from the interview recordings. The transcripts were thereafter printed and provided to the interviewers for verification of their alignment with the original meaning. Subsequently, the Chinese interview data was translated into English and submitted to a translator for verification. Inter-coder reliability assessments have been suggested to enhance reliability by evaluating concordance among several coders [102]. According to Miles and Huberman [103]’s guidelines for two researchers working together on qualitative analysis. Therefore, thematic analysis was completed by the researcher and teacher A (a lecturer in TESOL with more than ten years of teaching experience). The coding process follows the Open, Axial, and Selective Coding approaches by [104]. Codes were generated to satisfactory levels after the researcher and teacher A’ discussions and clarifications. Upon the conclusion of the three procedures, two Chinese experts in Chinese vocational EFL teaching field reviewed the coding process and provided suggestions concerning the emerging themes and sub-themes.

**Table 1. Coding example of interview transcripts**

| **Questions & Answers** | **Open coding** | **Axial coding** | **Selective coding (Sub-themes)** | **Themes** |
| --- | --- | --- | --- | --- |
| S1-Q3: Did you feel motivated to learn vocabulary in this CoI-based blended learning course?  A: *I think my motivation for learning is pretty good because, in this way, we can be more engaged. Then sometimes the teacher uses some encouragement methods. For example, every week the teacher evaluates the top three students in the system and rewards them. This makes us more motivated to learn. In addition, I think the Chaoxing Learning app is very practical and useful, there are so many functions and these functions brought us fun activities.* | 1)The teacher uses some encouragement methods which made the students more motivated to learn;  2)The Chaoxing Learning app is very practical and useful with many functions.  3)Chaoxing Learning app provides the students fun activities. | 1)Teachers take encouraging measures;  2)The platform has practical and useful functions;  3)Fun activities taken to motivate students; | 1)Confidence encouraging;  2)Useful functions;  3)Interest stimulating; | 1)Emotion;  2)Communication method; |
| S2-Q4: What do you think the strengths and weaknesses of vocabulary acquisition are in this CoI-based blended learning course?  *A: The disadvantage is that secondary school students are more or less unconscious, although they are usually very active in the classroom, there are inevitably a few students who take the opportunity to browse other websites when having class. Another disadvantage is that we have to do the homework on the mobile phone, and some of the answers can be found directly online. Some students will just copy and paste it over without using their brains at all. The advantage is that it can improve the enthusiasm of students because mobile phones have a youthful nature. For students of our age, being able to get along with mobile phones will make us very excited, and then those students who are sleeping can be effectively stopped.* | 1)A few students browsed other websites during the class;  2)Some students just copy and paste the answers online;  3)This CoI-based blended learning approach improved students’ enthusiasm;  4)Mobile phones made students excited. | 1)Students autonomous learning is not good;  2)Students self-control ability should be enhanced; | 1)Autonomous learning;  2)Self-control ability; | 1)Supervision; |
| S3-Q5: What do you think of the online and offline discussion in this CoI-based blended learning environment?  *A:I feel that in this blended learning environment, there is much more interaction between the teacher and the students and between the students themselves than in the traditional classroom. This mutual interaction is very helpful to enhance the relationship between the students and also between the teacher and the students. Students often discuss and solve problems proactively and enthusiastically online and offline together. They do not evade responsibility. Everyone participates in discussions actively, voluntarily, and proactively.* | 1)There is much more interaction between the teacher and the students and between the students themselves in this CoI-based blended learning environment;  2)Mutual interaction is very helpful in enhancing the relationship between the students and also between the teacher and the students;  3)Students often discuss and solve problems proactively and enthusiastically online and offline together. | 1)Interactions occurred among the teacher and students;  2)The relationship between the teacher and students was good;  3)Students solve problems actively. | 1)Effective communications;  2)Teacher-students, Students-students;  3)Solutions digging. | 1)Interaction;  2)Affective collection;  3)Meaningful learning |

- **Statistical Analysis:**

**Comment 1-**The use of paired and independent t-tests is appropriate for the pre/post comparisons. The reporting of effect sizes (Eta squared) is also commendable.
• Suggestions for Improvement:
Report confidence intervals for the effect sizes to better interpret practical significance.

**Author’s revision**

The author recalculated the effect size via Cohen’s d and Cohen’s d confidence interval.

**-P10**

Cohen’s d and the Confidence Interval were used to compute the effect size. The formula for Cohen’s d is:

Where: 1) *M_1_* and *M_2_* are the means of the two groups being compared. 2) *SD_1_* and *SD_2_* are the standard deviations of the two groups. The standard error of Cohen’s d was used to calculate the confidence intervals. The formula for the standard error is:

Where: *n_1_* and *n_2_* are the sample sizes of the two groups. The 95% confidence intervals for Cohen’s d were calculated using the standard error. The formula for the confidence intervals is:

CI = *d* ± 1.96 × *SE_d_*

Where: 1) *d* is the calculated Cohen’s *d*. 2) SE*d* is the standard error of Cohen’s *d*. 3) 1.96 is the z-score corresponding to a 95% confidence level (for a two-tailed test). Conversely, qualitative data were analyzed via thematic analysis.

**-P17**

According to [106], Cohen’s d values of 0.2, 0.5, and 0.8 are typically considered to represent small, medium, and large effects, respectively. The Cohen’s d effect size for this comparison was 1.09, with a 95% confidence interval of [0.92, 1.25], which indicates a large effect size, suggesting that it is 95% confident that the true effect size lies within this range. The wide interval, which does not include zero, further supports the conclusion that the CoI-based blended learning approach significantly enhanced students’ VTS compared to the traditional face-to-face method.

**-P18**

The Cohen’s d effect size for this improvement was 1.36, with a 95% confidence interval of [0.89, 1.82], indicating a large effect size and suggesting that it is 95% confident that the true effect size lies within this range. This substantial increase in VTS suggests that the CoI-based blended learning approach had a significant positive impact on students’ vocabulary acquisition.

**-P19**

The Cohen’s d effect size for this difference was 0.67, with a 95% confidence interval of [0.56, 0.77]. This indicates a medium effect, suggesting that it is 95% confident that the true effect size lies within this range. This interval, which excludes zero, provides strong evidence that the CoI-based blended learning approach effectively increased students’ VLM compared to the control group.

**-P20**

The Cohen’s d effect size for this improvement was 0.75, with a 95% confidence interval of [0.32, 1.18]. This indicates a medium effect, suggesting that it is 95% confident that the true effect size lies within this range. This result highlights the positive influence of the CoI-based blended learning approach on students VLM.

**Comment 2-**Address whether the data met the assumptions of normality (e.g., via Shapiro–Wilk tests) and discuss any steps taken if assumptions were violated.

**Author’s revision**

**-P17**

Thode [105] claims that the Shapiro-Wilk test is the optimal method for assessing normality in data when sample sizes are below 50. Upon analyzing the p-values in the Sig. column of the Shapiro-Wilk tests presented in Table 5, it is evident that all p-values exceeded 0.05. Therefore, the distributions of the Vocabulary pre-test and post-test scores, as well as the VLM pre-intervention and post-intervention scores for both the experimental and control groups, were normally distributed. The parametric paired samples t-test and independent samples t-test can be proceeded.

**Table 5. Normality test results for VTS and VLM measures by group**

| **Group** | **Item** | **Shapiro-Wilk** | | |
| --- | --- | --- | --- | --- |
|  |  | **Statistic** | **df** | **Sig.** |
| **Experimental group** | VTS-pre | 0.951 | 44 | 0.059 |
|  | VTS-post | 0.949 | 44 | 0.052 |
|  | VLM-pre | 0.97 | 44 | 0.297 |
|  | VLM-post | 0.958 | 44 | 0.111 |
| **Control group** | VTS-pre | 0.952 | 42 | 0.073 |
|  | VTS-post | 0.955 | 42 | 0.100 |
|  | VLM-pre | 0.984 | 42 | 0.818 |
|  | VLM-post | 0.979 | 42 | 0.641 |

**VTS: Vocabulary test scores; VLM:Vocabulary learning motivation;*

**Comment 3-**Although the effect sizes for VTS improvements (η² = 0.232) and VLM (η² = 0.092 and 0.279) are reported as “moderately weak,” a discussion of what these values imply in an educational context would be beneficial.

**Author’s revision**

**-P26-29**

-The author has recalculated the effect size by Cohen’s d. The discussion part has been rewritten as follows:

This study sought to examine the effect of a Community of Inquiry (CoI)-based blended learning approach on students’ Vocabulary Test Scores (VTS) and Vocabulary Learning Motivation (VLM) in Chinese secondary vocational EFL classrooms. The results demonstrated substantial enhancements in both VTS and VLM for students subjected to the CoI-based blended learning method in contrast to those in the conventional face-to-face learning setting. Furthermore, students exhibited favorable perceptions of this CoI-based blended learning approach. This study advances prior research by demonstrating how certain attributes of Teaching Presence (TP), Social Presence (SP), and Cognitive Presence (CP) affect students’ VTS and VLM in vocational blended learning contexts.

The findings from the first sub-research question indicated an approximately 9-point vocabulary gain with a large effect size. Chinese secondary vocational students generally start with vocabulary sizes 800-1,000 words beneath the national secondary education benchmark. Conventional vocabulary teaching methods have faced criticism for their excessive dependence on rote memorization and decontextualized word lists. Therefore, this significant enhancement is especially remarkable within the realm of Chinese secondary vocational education. The success of the VTS intervention can be systematically elucidated using the three components of the CoI framework. The TP component was implemented via meticulously crafted digital scaffolding that tackled essential issues in vocational EFL training. Consistent with previous studies [104,105,14,8], the instructor’s well organized lesson plans, incorporating pre-class previews, in-class exercises, and post-class reinforcement, guaranteed that students got scaffolded vocabulary training. The multimodal vocabulary presentation, which integrated video, text, and audio, boosted students’ involvement and facilitated students’ transition from rote memory to profound cognitive thinking [107]. Furthermore, interviewees indicated that the instructor’s feedback markedly enhanced their confidence. This corresponds with [108], indicating that the teacher’s role in facilitating discourse, delivering immediate feedback, and controlling group interactions assisted students in consolidating language. Social Presence was similarly vital to the success of the intervention, especially in tackling the motivating issues common in vocational EFL settings. This research emphasizes the critical role of fostering a collaborative online learning community to enhance students’ performance, consequently improving their engagement, consistent with the findings of [109,110,111]. This addressed as the principal impediment for vocational students: lack of motivation and engagement rather than cognitive incapacity. Interviews indicated that students had greater experiences of posing questions online compared to face-to-face classroom settings. That was because the CoI-based blended learning environment enabled students to share feelings, challenges, and achievements, thereby cultivating a supportive environment [27]. Such feeling of community is vital for enabling students to feel welcomed, supported, and accepted, hence increasing their VTS. These conclusions are consistent with the findings of [112-114]. CP was developed through well-organized exercises that fostered advanced engagement with vocabularies. According to [115], CP improves learner interest and active participation in online EFL discussions. Community members were urged to participate in peer interaction, problem-solving, inquiry, proposing solutions, and collaboration, thereby cultivating a flexible learning environment. Problem-based scenarios necessitated the contextual application of newly acquired vocabulary, fostering the desirable difficulty that has been demonstrated to improve long-term memory [116]. Metacognitive reflection exercises, in which students recorded and examined their vocabulary acquisition techniques, leveraged recent findings indicating that explicit strategy instruction can significantly aid vocational learners [117]. Furthermore, the components of the flipped classroom facilitated enhanced cognitive engagement during in-person sessions, aligning with recent research indicating that prior digital exposure liberates cognitive resources for advanced application in face-to-face classrooms [118].

The second sub-research question indicated a notable increase in the experimental group’s VLM score of a 0.38-point improvement together with a medium effect size. This transition, as seen by [119], indicates a progression of students’ VLM from a Moderate level to a Motivated level. The enhancement of students’ VLM scores can be systematically elucidated by the CoI framework’s three presences which fostered students’ fundamental psychological needs of autonomy, competence, and relatedness [48]. First, by integrating well-crafted decision-making frameworks into blended learning, TP promoted students’ autonomy. In this study, TP offered autonomous support via three processes, in accordance with [120]: 1) Self-directed vocabulary modules enabling students to follow their own learning pace, 2) optional challenge tasks for advanced learners, and 3) customized learning path generated by the Chaoxing Learning APP algorithm. Competence was developed through the immediate feedback from the teacher and system. Recent neurocognitive studies indicate when learners proficiently acquire new vocabularies, their brain’s reward circuitry can be stimulated [121]. The varied assessment techniques in accordance with [122], such as peer evaluations and digital badges, established visible competence progression. It is essential for sustaining motivation in vocational students who frequently question their language learning capabilities. This study confirmed the findings of [123] that SP significantly affects learners’ EFL motivation, engagement, and overall learning outcomes in blended or online language learning environments. SP markedly improved relatedness by organized peer interactions. Collaborative contexts and peer interactions enhanced competence development through modeling, feedback, and shared challenges. This consequently enhanced VLM, as learners who perceived themselves as competent and socially supported demonstrated increased persistence [48]. In addition, digitally-mediated cooperation can overcome the social anxiety commonly found in vocational classrooms. CP enhanced all three psychological demands via cognitively stimulating language exercises. In alignment with [28,124], this CoI-based integrated learning environment offers a forum for students to exchange their perspectives on vocabulary acquisition and engage in critical discourse. Students can express difficulties, seek knowledge, comprehend essential issues through the integration of existing information, and subsequently formulate solutions to tackle those challenges. Autonomy was augmented by problem-based learning scenarios that permitted several solution pathways. The CP features unexpectedly enhanced relatedness, as students identified common difficulties and solutions during collaborative meaning-making activities [125]. Tasks were calibrated to balance challenge and skill, avoiding both oversimplification and excessive difficulty. These carefully scaffolded exercises fostered students’ competence. Metacognitive reflection exercises further targeted vocational students’ self-efficacy, with recent evidence showing such activities rebuild confidence in struggling learners. This sense of ownership and participation motivates students to engage more actively in their studies, in accordance with [81].

The results from the second research question indicated that students have a favorable and preferred perception of this CoI-based blended learning approach. The advantages of this CoI-based blended learning approach were revealed nine themes and 27 sub-themes from TP, SP and CP. In line with [111], when educators are adept at using technology resources, students’ learning experiences are markedly enhanced. Align with [126, 127], professional instruction, prompt feedback, varied evaluations, and consideration of students’ emotions cultivated students’ positive perceptions of TP. Furthermore, in accordance with [82] trust was fostered through collaboration, communication, and dialogue, which enhanced the development of confidence in self-expression. Students and teachers can openly discuss and express their authentic feelings through various channels, including online forums on the Chaoxing Learning App and face-to-face interactions. All these enable students’ positive perceptions of SP. Moreover, the metacognitive components of this CoI-based blended learning environment effectively cultivated students’ curiosity, strategic awareness and problem-solving skills [128], the enhanced CP helped address the recognized deficiency in the learning skills of vocational students.

Notwithstanding the favorable opinions expressed by the students, the implementation of this CoI-based blended learning approach in practical teaching also poses challenges. For example, students struggled to maintain sustained concentration during class. They also faced difficulties resisting distractions, particularly from mobile phone usage. Additionally, many found it challenging to avoid off-task conversations with peers. These issues were linked to underdeveloped self-regulation and autonomy skills. Therefore, students suggested that the teacher should give more supervision during the learning process in the future. In the same way, past studies [129-131] all noted that structured and supportive learning environments contribute to better academic resilience. Teachers ought to provide guiding roles by offering helpful feedback, impartial observations, and constructive views during tasks. Furthermore, students noted that the Chaoxing Learning App is inadequate for facilitating CoI-based blended learning. Future learning processes require a variety of supplementary digital tools that are engaging, valuable, user-friendly, and effective for interaction with instructors, peers, and content. In this way, instructors are able to develop more dynamic, efficient, and equitable language learning experiences [90]. Besides, some students believed that certain functions of the Chaoxing Learning App might be enhanced to increase its convenience. In the future, it should facilitate students’ access to and download of courseware and instructional materials from any location at any time, while also allowing them to structure their own learning process. To address this problem, [73] suggested that the use of AI-driven platforms can facilitate more tailored and adaptive assessment experiences that correspond with the requirements of a contemporary, blended educational framework. What’s more, the CoI-based blended learning approach also faces challenges in low-tech vocational settings. Dindar et al. [132] suggestion of using fundamental mobile platforms to act as alternatives to more advanced systems can be considered.

- **Results and Discussion**

**Comment 1-**Results Presentation:
• The quantitative results are clearly presented in tables with appropriate statistical values. However, the narrative could be improved by integrating the numbers with practical implications.
• For example, in Table 4.5, you report that experimental group VTS increased from 15.64 to 25.43 (t(43) = -8.627, p = 0.000). The discussion should explain what a 9-point increase on a 40-point scale means for student learning outcomes.

**Author’s revision**

**-P26-29**

-The author has rewritten the discussion part. Please refer to the above mentioned text in section-Statistical Analysis-comments 3 or the tracked manuscript.

**Comment 2-**Qualitative Findings:
• The qualitative findings are detailed, with themes related to Teaching Presence, Social Presence, and Cognitive Presence well described. Consider adding a summary table that links themes with representative quotes to improve clarity.

**Author’s revision**

**-P22**

Table 14. Themes related to teaching presence

| **Teaching Presence** | **Guidance** | B) **Professional**   **Instruction** | *I could feel that the teacher had prepared her class more carefully in this blended learning environment. The teacher incorporated a lot of* ***professional knowledge*** *related to the subject of English. It is not as casual as traditional teaching.*  (S5) |
| --- | --- | --- | --- |
|  |  | C) **Timely**   **Feedback** | *We used to have to wait a long time for the teacher to finish correcting our assignments and papers after exams, but with the blended learning model, we can know where we* ***went wrong in time*** *and make targeted corrections.*  (S10) |
|  |  | D) **Diverse**   **Evaluations** | ***Peer assessment*** *is one of my favorite classroom activities, and whenever the teacher distributes students work, the classroom can be somewhat active.*  (S4) |
|  | **Emotion** | B) **Behaviour**   **respecting** | *In traditional education, teachers may be reluctant to ask students with low grades to answer questions. But in the blended learning approach, independent of our grades, we can engage in quizzes, brainstorming, topic discussions, group projects, etc. I feel that everyone* ***received adequate attention****.*  (S9) |
|  |  | C) **Confidence**   **encouraging** | *In the past, I was afraid of answering the teacher’s questions because I was afraid that I would make mistakes, but in this blended learning environment, I am active to challenge myself because it is really* ***encouraging from the teachers side****.*  (S12) |
|  |  | D) **Diverse**   **Interest stimulating** | *In this blended teaching mode, everyone is more active and* ***interested especially in learning vocabulary****, and then, in traditional teaching like in junior middle school, the teacher usually follows the book and speaks in a very rigid way, and then you tend to get distracted in class.*  (S3, S4, S6) |
|  | **Supervision** | D) **Autonomous**   **learning** | *As secondary school students are more or less unconscious, although they are usually very active in the classroom, there are inevitably a few students who take the opportunity to* ***browse other websites*** *when having class.*  (S2) |
|  |  | E) **Self-control**   **ability** | *Sometimes group members would* ***browse other websites*** *not related to the class, therefore, it is necessary for us to gain the teachers supervision.*  (S8) |
|  |  | F) **Diverse**   **Continuous grit** | *I feel that learning English is a long-term process, and because our classmates have all been rather disinterested in the past, I was concerned that they would find it* ***difficult to continue*** *once the novelty wore off.*  (S5) |

**-P23**

Table 15. Themes related to social presence

| Teaching Presence | **Interaction** | **A) Cooperative Collaboration** | *I could feel that the teacher had prepared her class more carefully in this blended learning environment. The teacher incorporated a lot of* ***professional knowledge*** *related to the subject of English. It is not as casual as traditional teaching.*  (S5) |
| --- | --- | --- | --- |
|  |  | **B) Healthy Competition** | *We used to have to wait a long time for the teacher to finish correcting our assignments and papers after exams, but with the blended learning model, we can know where we* ***went wrong in time*** *and make targeted corrections.*  (S10) |
|  |  | **C) Effective Communication** | ***Peer assessment*** *is one of my favorite classroom activities, and whenever the teacher distributes students work, the classroom can be somewhat active.*  (S4) |
|  | **Affective collection** | **A)Teacher-**  **students** | *In traditional education, teachers may be reluctant to ask students with low grades to answer questions. But in the blended learning approach, independent of our grades, we can engage in quizzes, brainstorming, topic discussions, group projects, etc. I feel that everyone* ***received adequate attention****.*  (S9) |
|  |  | D) **Students-**   **students** | *In the past, I was afraid of answering the teachers questions because I was afraid that I would make mistakes, but in this blended learning environment, I am active to challenge myself because it is really* ***encouraging from the teachers side****.*  (S12) |
|  |  | E) **Community-**   **community** | *In this blended teaching mode, everyone is more active and* ***interested especially in learning vocabulary****, and then, in traditional teaching like in junior middle school, the teacher usually follows the book and speaks in a very rigid way, and then you tend to get distracted in class.*  (S3, S4, S6) |
|  | **Communication method** | **A) Diversify tools** | *I think Chaoxing Learning app is very practical and useful, there are so many functions and these functions brought us fun activities.*  (S1) |
|  |  | **B) Convenient platform** | *I think the function of* ***submitting assignments*** *and* ***having tests*** *in Chaoxing Learning App is very good.*  (S2) |
|  |  | **C) Useful functions** | *Hope in the future, more digital tools can be introduced to our English class.*  (S8) |

**-P25**

Table 16. Themes related to social presence

| Teaching Presence | **Strategy** | **A) Using Digital Resources** | *I never knew before that there were so many rich* ***extra-curricular resources*** *for us secondary school students. In the past, when we were taught traditionally, we only listened to the teacher and if we didn’t know what to do, we put it aside, but in the blended teaching mode, the teacher provided us with many learning websites and links. if we dont understand what we know in class, we can consolidate it again and again in online classes until we learn it.*  (S12) |
| --- | --- | --- | --- |
|  |  | **B) Using Novel Tools** | *I love* ***dubbing*** *the most. I used to be very shy about speaking English, but through the* ***funny animations*** *in dubbing, Ive forgotten about being embarrassed to speak English. We can actually learn it when we speak it.*  (S6) |
|  |  | **C) Effective Instructions** | *Although the effect of cooperative learning is very good, sometimes we are not very clear about some knowledge points. It will be much better if the teacher* ***guides*** *us.*  (S7) |
|  | **Course Approach** | **A) Immense Learning** | *The role-playing in the blended learning approach allows us to* ***immerse ourselves*** *in it as if we were the protagonists.*  (S6) |
|  |  | **B) Flipped Classroom** | *This blended learning mode is helpful in overcoming students’ shyness and fear through* ***individual presentations*** *and* ***group presentations****.*  (S11) |
|  |  | **C) Learning Communities** | *The* ***learning community*** *is just like a family, we just discuss and solve the problems together. It is helpful for me to better recognize the knowledge.*  (S2) |
|  | **Meaningful Learning** | **A) Curiosity Arousal** | *In this CoI-based blended learning, the teacher would often set us up with suspense to* ***stimulate our curiosity****. I think it is very important for us to learn new knowledge.*  (S10) |
|  |  | **B) Solutions Digging** | *In the process of finding the* ***solutions to the problems****, I believe I gained knowledge.*  (S6) |
|  |  | **C) Ideas Exploration** | *When the community work together, some* ***new ideas*** *come out.*  (S4) |

**Comment 3-**Discussion of Findings:
• The discussion appropriately links the findings back to the CoI framework. However, it would benefit from a more critical comparison with previous studies. For example, discuss how your findings align with or differ from those reported by, say, Chen et al. (2021) or Garrison (2017), and what this means for EFL pedagogy.
• Additionally, explicitly discuss the practical significance of the blended learning intervention in terms of potential scalability in vocational settings.

**Author’s revision**

**-P26-29**

-The author has rewritten the discussion part. The practical significance of the blended learning intervention in terms of potential scalability in vocational settings have been discussed. Please refer to the above mentioned text in section-Statistical Analysis-comments 3 or the tracked manuscript.

- **Language and Style**

**Comment 1-**Several sentences are lengthy and could be streamlined for clarity. For example, revise:
“The teacher provided detailed feedback and systematic information about their presentation, allowing students to actively engage in discussions and develop their understanding through peer and teacher interaction.” to a more concise version.
• Check for minor typographical errors (e.g., “is there any improvement of their VTS and VLM” should be “if there is any improvement”).

**Author’s revision**

-The author has checked the lengthy sentences and simplified them. Typographical errors have been checked throughout the manuscript. eg:

**-P1**

“The mechanical traditional way of teaching and their lacking amount of vocabulary toward the target language often demotivate them in learning.”

>“The mechanical traditional way of teaching and their lack of vocabulary toward the target language often demotivate them in learning.”

**-P6**

“Students were frequently provided with voluminous paper-based vocabulary books and subjected to the laborious process of passive learning.”

>“Students have to rely on extensive paper-based vocabulary exercises, a method that often reduces engagement and limits contextualized learning opportunities. ”

**-P7**

“With this approach, students are offered a new learning experience and able to interact with digital platforms, extending the variety of available resources and addressing the needs of different learning styles with a combination of texts, images, audio, and videos (Tosun, 2015)”.

>“This approach offers students an entirely novel educational experience. By interacting with digital platforms, they may access more materials and use multimedia such as texts, photos, audio, and videos to meet different learning styles [75].”

...please refer to the tracked manuscript.

- **Formatting**

**Comment 1-**Tables and figures should be uniformly formatted. Ensure that table captions are clear and that all abbreviations (e.g., VTS, VLM) are defined in each table.

**Author’s revision**

-All abbreviations have been defined in each table, please refer to the tracked manuscript.

Tables and figures have been uniformly formatted, please refer to the tracked manuscript.

**Comment 2-**In the literature review, ensure that all in-text citations are consistent and correctly formatted.

**Author’s revision**

-The author has checked all the in-text citations, please refer to the tracked manuscript.

**Comment 4-**“Over the past few years, blended learning has become increasingly popular …”
Suggest revising to:
“In recent years, the adoption of blended learning has surged, owing to its flexibility and the integration of digital tools that foster both independent and collaborative learning (Cronje, 2020; Polakova & Klimova, 2022).”

**Author’s revision**

**-P2**

The author has revised it as suggested.

“In recent years, the adoption of blended learning has surged, owing to its flexibility and the integration of digital tools that foster both independent and collaborative learning [1,5,6]. ”

**Comment 5-**“The Vocabulary Test compromises 40 multiple-choice questions…”
Correct to:
“The Vocabulary Test consists of 40 multiple-choice questions…”

**Author’s revision**

**-P8**

-The author has revised it as suggested. See the instruments- Vocabulary pre-test and post-test part.

...“In this study, a researcher-developed Vocabulary Test was employed for both the pre-test and post-test. The test consisted of 40 multiple-choice questions, each valued at one point, yielding a maximum score of 40”. ...

**Reviewer #3 comments**

- **Language and Style**

**Comment 1-**“The lacking amount of vocabulary…” → “The lack of vocabulary…”

**Author’s revision**

**-P1**

“The mechanical traditional way of teaching and their lack of vocabulary toward the target language often demotivate them in learning”.

**Comment 2-**“their VTS and VLM” → better to spell out at first mention in each section.

**Author’s revision**

-The author has added the full name of VTS and VLM at first mention in each section. Please see the tracked manuscript highlighted parts.

**Comment 3-**Verb tense consistency in the discussion and methodology needs attention.

**Author’s revision**

-The author has corrected the tense throughout the manuscript. Please see the manuscript.

- **Data Availability**

**Comment 1-**Though you mentioned data is available upon request, PLOS encourages open-access sharing of datasets. Please consider uploading your anonymized data to a repository like OSF and linking it in your manuscript.

**Author’s revision**

-The data has been added as supplementary files with the manuscript.

- **Theoretical Implications**

**Comment 1-**The discussion would benefit from deeper theoretical integration—for example, explicitly linking student motivation to the CoI's presences, or citing motivational frameworks like Self-Determination Theory to anchor the findings.

**Author’s revision**

-the author has rewritten the discussion part. The second sub-research question linked students’ motivation to the CoI's presences, and also cited the Self-Determination Theory to anchor the findings.

**-P27-28**

**Discussion**

The second sub-research question indicated a notable increase in the experimental group’s VLM score of a 0.38-point improvement together with a medium effect size. This transition, as seen by [119], indicates a progression of students’ VLM from a Moderate level to a Motivated level. The enhancement of students’ VLM scores can be systematically elucidated by the CoI framework’s three presences which fostered students’ fundamental psychological needs of autonomy, competence, and relatedness [48]. First, by integrating well-crafted decision-making frameworks into blended learning, TP promoted students’ autonomy. In this study, TP offered autonomous support via three processes, in accordance with [120]: 1) Self-directed vocabulary modules enabling students to follow their own learning pace, 2) optional challenge tasks for advanced learners, and 3) customized learning path generated by the Chaoxing Learning APP algorithm. Competence was developed through the immediate feedback from the teacher and system. Recent neurocognitive studies indicate when learners proficiently acquire new vocabularies, their brain’s reward circuitry can be stimulated [121]. The varied assessment techniques in accordance with [122], such as peer evaluations and digital badges, established visible competence progression. It is essential for sustaining motivation in vocational students who frequently question their language learning capabilities. This study confirmed the findings of [123] that SP significantly affects learners’ EFL motivation, engagement, and overall learning outcomes in blended or online language learning environments. SP markedly improved relatedness by organized peer interactions. Collaborative contexts and peer interactions enhanced competence development through modeling, feedback, and shared challenges. This consequently enhanced VLM, as learners who perceived themselves as competent and socially supported demonstrated increased persistence [48]. In addition, digitally-mediated cooperation can overcome the social anxiety commonly found in vocational classrooms. CP enhanced all three psychological demands via cognitively stimulating language exercises. In alignment with [28,124], this CoI-based integrated learning environment offers a forum for students to exchange their perspectives on vocabulary acquisition and engage in critical discourse. Students can express difficulties, seek knowledge, comprehend essential issues through the integration of existing information, and subsequently formulate solutions to tackle those challenges. Autonomy was augmented by problem-based learning scenarios that permitted several solution pathways. The CP features unexpectedly enhanced relatedness, as students identified common difficulties and solutions during collaborative meaning-making activities [125]. Tasks were calibrated to balance challenge and skill, avoiding both oversimplification and excessive difficulty. These carefully scaffolded exercises fostered students’ competence. Metacognitive reflection exercises further targeted vocational students’ self-efficacy, with recent evidence showing such activities rebuild confidence in struggling learners. This sense of ownership and participation motivates students to engage more actively in their studies, in accordance with [81].

**P4-6**

**Literature review- Community of Inquiry and learning motivation**

Learning motivation influences students’ learning behaviors and guides them towards particular academic objectives [38]. Self-determination theory (SDT) posits that an individual’s desire to learn during an activity is contingent upon the extent to which the learning environment fulfills the three fundamental psychological requirements of autonomy, competence, and relatedness [39]. According to [40], these three fundamental psychological needs are significantly correlated with the three dimensions of the Community of Inquiry (CoI) framework: Teaching Presence (TP), Social Presence (SP), and Cognitive Presence (CP).

Comprehensive studies have established a substantial correlation between Teaching Presence (TP) and students’ motivation [41], especially within the context of Self-Determination Theory (SDT). Autonomy support is augmented when educators employ structured instructional frameworks, offer avenues for students’ choice, and promote self-directed learning [42]. By providing timely, substantive feedback and exhibiting passion, educators create an environment that empowers students to take initiative, thus nurturing intrinsic motivation [43]. Moreover, active listening, empathy, and personalized support enhance students sense of agency, consistent with autonomy as a fundamental catalyst for engagement [44]. Secondly, TP enhances competency via constructive feedback, explicit expectations, and scaffolded learning experiences [45]. When educators create engaged and intellectually stimulating sessions, students cultivate mastery beliefs and self-efficacy [46]. A well-organized educational setting, along with formative evaluations, allows students to monitor their advancement, hence enhancing their perceived competence [47]. Ultimately, TP enhances relatedness by cultivating a secure, inclusive, and cooperative classroom environment. Educators who demonstrate positive regard, accessibility, and emotional support foster a sense of value and connection among students [47]. The presence of social interaction in education, facilitated by responsive communication and community-building initiatives, improves relationships among peers and between instructors and students, thereby satisfying students demand for belonging [44]. This sense of connection is essential for enduring motivation, since it reduces disengagement and enhances emotional well-being [48].

Empirical research indicates that Social Presence (SP) is essential for satisfying learners’ psychological needs for autonomy, competence, and relatedness, thus augmenting intrinsic motivation and academic engagement [49]. First, SP enhances autonomy by promoting student-centered learning environments in which learners engage actively in decision-making and collaborative activities [50]. Students participating in peer discussions, group projects, or self-directed activities gain increased control over their learning, corroborating SDT’s claim that autonomy-supportive environments augment motivation [48]. Moreover, interactive and dialogic pedagogical approaches, including peer evaluations and collaborative problem-solving, enhance learners’ feeling of agency, diminishing dependence on external regulation and fostering self-determined behavior [49]. Second, the concept of competence is enhanced by SP, facilitating meaningful interactions that affirm learners’ capacities and support skill development [51]. Collaborative exercises, including group discussions and peer feedback sessions, enable students to assess their comprehension, enhance their knowledge, and obtain constructive feedback from peers and instructors. These interactions enhance subject-matter expertise and foster metacognitive and self-regulatory skills, vital for enduring academic achievement [52]. When educators facilitate supportive and organized social interactions, students cultivate enhanced self-efficacy, viewing themselves as competent participants in the learning community [53]. The most immediate impact of SP is on relatedness, since it fulfills the fundamental human need for connection and belonging [54]. A strong sense of community in educational settings enhances students emotional engagement and reduces feelings of isolation [55]. Small-group discussions and cooperative learning tasks, foster students’ trust and mutual support [55]. This interactive and socially enriched environments augment intrinsic motivation by validating students identities as legitimate participants in academic discourse [56].

Studies have repeatedly shown a significant beneficial correlation between Cognitive Presence (CP) and students’ motivation [57, 58]. CP promotes autonomy by facilitating self-directed inquiry and intellectual exploration [59]. Students participate in higher-order cognitive processes, including critical reflection and knowledge application, achieve enhanced congruence between their learning activities and personal values [60]. CP’s problem-solving aspect enables learners to exercise autonomous judgment, make significant decisions regarding their learning methodologies, and cultivate a sense of ownership over their educational experience [61]. The correlation between CP and competency is well-documented in the literature. By engaging consistently in critical discourse and using knowledge practically, students build subject-matter competence and metacognitive skills [62]. The iterative process of inquiry, reflection, and problem-solving intrinsic to CP offers ongoing chances for students to showcase and enhance their abilities [63]. When educational settings offer explicit learning objectives and formative assessment opportunities, students report increased emotions of competence and self-efficacy [64]. The predominantly cognitive aspect of persistent critical thinking and knowledge production also promotes relatedness via collaborative meaning-making [59]. The social aspect of CP arises during collaborative problem-solving tasks in which students participate in collective reflection and analysis [65]. This shared cognitive endeavor fosters a sense of intellectual community, fulfilling students need for belonging in academic environments [62]. The reflective elements of CP allow students to relate their learning experiences to wider social contexts, so augmenting their sense of purpose and affiliation with the learning community [57].

- **Limitations and Future Research**

**Comment 1-**You identified gender imbalance and sample size/location as limitations. You might further suggest longitudinal studies to explore retention of vocabulary and motivation over time, or comparative studies across disciplines.

**Author’s revision**

**-P30**

“First, according to the characteristics of vocational majors, a limitation in terms of the gender imbalance among students exists in this study. Future studies could purposefully select gender-balanced cohorts from a variety of vocational disciplines to examine the effectiveness of the CoI framework in order to address these discrepancies. Although the sample’s gender imbalance (81.8% male) reflects the demographic reality of Chinese vocational schools, it restricts the possibility to assess the potential influences of gender differences on CoI-based blended learning. Therefore, research on the effectiveness of this approach should focus on socioeconomic or gender-based demographics. Second, due to time constraints, this study was designed as a quasi-experiment with a duration of 13 weeks, to enhance analytical rigor, future research should incorporate longitudinal methodologies to explore causal relationships more robustly and validate findings on vocabulary retention and motivation. ”

- **Figures & Framework (Fig 5.1)**

**Comment 1-**Your conceptual framework is a useful synthesis. To enhance its practical value, consider explaining how teachers might operationalize each element in low-tech or resource-constrained classrooms.

**Author’s revision**

-The author has added a guideline of this framework as a supplementary file to explain it.

**-P29**

What’s more, the CoI-based blended learning approach also faces challenges in low-tech vocational settings. Dindar et al.’s [132] suggestion of using fundamental mobile platforms to act as alternatives to more advanced systems can be considered. For example, in high-tech regions, AI-powered tutoring system and VR simulations can be applied; however, in low-tech areas, We-chat group and Tencent QQ group can be used instead.

Table 1

Guidelines for conducting the CoI-based blended learning (Steps of designing learning procedure)

| **Procedure** | **Approach** | **Descriptions** |
| --- | --- | --- |
| **Preparations** | **--** | Before classes began, students were divided into distinct communities based on their performances and personalities. Online learning forums were also created on the ChaoXing Learning Platform according to the communities. In each community, students were allocated roles such as group leader, technical recorder, designer, and connector, and were responsible for a variety of learning-related tasks. |
| **Before class** | **Online** | **Information collection:** Individual and group assignments were assigned to students, who then met and discussed using the ChaoXing Learning Platform on blended distributed terminals to share the required and collected information for the class. |
|  |  | **Personal & group tasks:** The teacher provides various learning contents through the network, students complete their individual and group assignments using various digital media, such as their smartphones and laptops, at their preferred time, space, and speed and upload them to the ChaoXing Learning Platform according to the requirements of the pre-class tasks issued by the teacher. |
|  |  | **Discussions:** Students meet and discuss to share the collected information needed for the class and upload the required documents to the ChaoXing Learning Platform. |
|  |  | **Problems proposing:** Discuss and propose the problems they were facing when doing the pre-class individual and group assignments. |
| **During class**  **(face-to-face)** | **Online + offline** | **Presentations & lectures:** Volunteers and randomly chosen students by the ChaoXing Learning Platform were asked to present their work, and teachers gave them constructive and specific feedback. |
|  |  | **Discussions:** Students in a community or inter-communities together with the teacher discuss and explore the knowledge. |
|  |  | **Summaries:** The teacher summarizes the knowledge of the present class, emphasizing teaching key points and teaching difficult points. |
|  |  | **Evaluations:** Diversification of evaluations was done by students’ self-evaluation, inter-student evaluation, teacher evaluation, and automatic evaluation by the ChaoXing Learning Platform. |
| **After class** | **Online** | **Knowledge reflecting:** Students reflect on their strengths and weaknesses in the lesson’s knowledge based on their understanding, exploration, and the teacher’s summaries. |
|  |  | **Personal & group assignments:** Consolidation and deepening of knowledge through individual and group assignments after class based on what has been learned. |
|  |  | **Knowledge integrating:** Use diagrams, mind maps, knowledge comparisons, and knowledge points to organize and integrate what have learned in class. Further knowledge application, problem analysis, and solution exploration. |
|  |  | **Problems solving:** Through students’ self-exploration, peer discussion, and teachers’ explanation, eventually lead to the resolution of the confusing questions and problems. |

Table 2

Guidelines for conducting the CoI-based blended learning (Measures should be taken during learning procedure)

| **CoI elements** | **Theme** | **Descriptions** |
| --- | --- | --- |
| **Teaching presence** | **Guidance** | **Professional instructions:** Teachers need to comprehend the theory, belief, principle, and idea underlying teaching activities or methods. They need to master the relevant professional knowledge of teaching, such as teaching environment, curriculum, teaching design, teaching mode and method, classroom management, teaching evaluation, and teaching ability of information technology. Students’ needs, qualities, differences, and motivations need to be understood as well. |
|  |  | **Timely feedback:** The feedback should be expeditious, immediate, constructive, multiple, empathetic, critical, and original. Provide timely feedback on students’ in-class participation, correctness of their performances, online and offline learning scoring, and emotions. Feedback should be made to let students be involved in the classroom, learn more about their capabilities, and maintain their self-confidence. Provide students with the direction they require to achieve their objectives. |
|  |  | **Diversify evaluations:** To ensure the authenticity, objectivity, and real-time of the entire academic evaluation, an evaluation system that combines teacher evaluation, peer evaluation, and self-evaluation; online evaluation and offline evaluation; initial evaluation and summative evaluation; dynamic evaluation and static evaluation; standard regular evaluation and random flexible evaluation should be designed. |
|  | **Emotion** | **Behavior respecting:** Teachers respond to and appreciate students’ behaviors by trusting, supporting, smiling, making eye contact, employing calming postures, having a great sense of humor, being kind, using a decent tone, and sharing positive emotions. |
|  |  | **Confidence encouraging:** Encourage, praise, and reward acceptable, learning-oriented behaviors among students. Produce positive psychological hints to boost their self-confidence, and cohesion, reduce their anxiety, and encourage them to be courageous in expressing their opinions. |
|  |  | **Interest stimulating:** Create a fun and playful environment that enhances student’s motivation and learning outcomes by appealing to their interests. Motivate and stimulate their desire to learn and seek more knowledge. Allow students to focus more on their work and eliminate monotonous lecturing routines by using authentic and interesting materials and situations. |
| **Teaching presence** | **Supervision** | **Autonomous learning:** Enhance students’ capacity for self-directed learning, including self-monitoring and self-reflection. Teachers serve as mentors to students, assisting them in identifying learning objectives, defining content and progressions, selecting methods and strategies to be used, monitoring the acquisition process, and analyzing what has been learned. Help students create realistic goals so that when they achieve them. Let students feel a sense of achievement and develop the ability to learn on their own. |
|  |  | **Self-control ability:** This includes strong self-management skills, the ability to concentrate in class, refraining from cell phone use, distraction, and gossiping with classmates. The ability to accomplish group and individual assignments assigned by the teacher self-consciously. |
|  |  | **Continuous grit:** Students own perseverance, ambition, and energy for long-term objectives, and have a clear and profound understanding motivations and goals of their actions. Be capable of regulating their emotions in response to setbacks and failures, controlling their actions, and remaining unfazed and unruffled. |
| **Social presence** | **Interaction** | **Cooperative cooperation:** Provide a safe and comfortable classroom environment and avoid using threats. Encourage students to become active, imaginative, critical, and accountable in order to achieve the team’s objective. Let students participate in the learning experience in the entirety and effective group discussion while working in groups to provide a positive experience for every participant. |
|  |  | **Healthy competition:** In a healthy competition environment, as opposed to individuals trying to outperform one another, students work to achieve organizational and common objectives. Friendship comes before competition. |
|  |  | **Effective communications:** Construct communities of blended learning among instructors and students. Create a classroom environment that is beneficial, engaging, interactive, and pleasurable. Encourage synchronous and asynchronous discussion, effective communication, and benign interaction as well as questioning, responding, and commenting among students. |
|  | **Affective collection** | **Teacher-students:** Teachers should be able to foster an environment in which students can interact freely and safely. Relationships between teachers and students in the classroom should be characterized by respect, enjoyment, mutual trust, and comprehension. |
| **Social presence** | **Affective collection** | **Students-students:** Positive relationships between group members to boost social interaction, individual accountability, and group competence. Learn to be tolerant and empathetic, to care for others, to praise others, to respect and accept others, and to manage conflicts between classmates. |
|  |  | **Community-community:** Strong community spirit, prompt to assist, share resources and information, and respond to inquiries. Positive and effective interactions, active learning practices, knowledge, ideas, experience, and learning products are valued and shared among communities. |
|  | **Communication method** | **Diversify tools:** Apply digital technologies, such as computer-assisted language learning (CALL) or mobile-assisted language learning (MALL), digital games, learning management systems (LMS), learning platforms, and other digital tools such as Google Form, Socrative, Kahoot!, Quizizz, Quizlet, Youdao vocabulary learning app, Wechat those have the potential to enhance teaching and learning. |
|  |  | **Convenient platform:** When implementing the CoI-based blended learning, a user-friendly platform, such as the ChaoXing Learning Platform, Rain Classroom, Ke Tang Pai, Tencent Meeting, Google Classroom, Embodo, etc., can be used. |
|  |  | **Useful functions:** Functions such as Sign-in, Information sharing, Competitive answering, Vote, Quiz, Discussion, Random candidate, Assign homework, Class report, Big data analysis, Evaluation, and E-learning design for effective interactions between teachers and students or among students. |
| **Cognitive presence** | **Strategy** | **Digital resources:** Make full use of online digital resources like Chinese University MOOC, Smart VET, Secondary Teaching Resources Website, Technical Education Website, Chongqing Cloud Platform, and resources in the ChaoXing Learning Platform. |
|  |  | **Novel tools:** Using new digital teaching tools that combine text, sound, and images to enrich classroom teaching, and improve the teaching quality of teachers and the learning efficiency of students. |
|  |  | **Effective instructions:** Excellent instruction should be provided. Curriculum, books, software, and other instructional materials should make sense to students. Make them engage and feel easy to apply. The objectives of each course should be specified in the instructions with varying prior knowledge. Instructions should also include new subject matter, simple language, vivid images, and examples. |
|  | **Course approach** | **Immense learning:** A form of contextualized language learning in which the learning process is situated in a diversity of virtual or actual language contexts. In addition to the interaction with the teacher making the class vivacious, active, and challenging, the students are fully engaged and aware of the learning and processes taking place throughout the entire class period. |
|  |  | **Flipped classroom:** Classes are held in digital environments that promote the development of students’ competencies through cooperative learning, problem-solving, and critical thinking. An emphasis is additionally put on dynamic interaction and the transfer of knowledge using digital tools in a collaborative and idea-sharing spirit. Students learn various topics separately utilizing video tutorials before applying their knowledge in class by working on practical exercises. Students are encouraged to learn alone and in groups, which facilitates the exchange of learning content throughout communities and, as a result, improves students’ problem-solving abilities. |
|  |  | **Learning communities:** Students allocate to small groups to master academic content. Classroom discussions, presentations, and group projects are progressively transformed into collaborative learning through the use of technology. |
|  | **Meaningful learning** | **Curiosities arousal:** Piquing students’ motivation and curiosity about the material to be taught. For example, use surprising demonstrations, the comparison of subjects to students’ own lives, or allow students to find out information by themselves. |
|  |  | **Solutions digging:** Encourage students to establish and reinforce meaning by having ongoing discussions and reflections. Encouraging critical thinking in a community of students through emotional, interactive, and coherent responses to help reach cognitive learning objectives. |
|  |  | **Ideas exploration:** Sharing the knowledge and information gained through group discussions. Encourage critical thinking, problem-solving, and new ideas-creating abilities among students. |
